# Supplementary material for: ZEB1/NuRD complex suppresses TBC1D2b to stimulate E-cadherin internalization and promote metastasis in lung cancer
Source: Nat Commun. 2019 Nov 12;10:5125. doi: 10.1038/s41467-019-12832-z (PMC6851102; doi:10.1038/s41467-019-12832-z)
Supplement: Supplementary file 1 — Supplementary Information [file 41467_2019_12832_MOESM1_ESM.pdf]

Supplementary Figure 1 (A) Relative mRNA expression of ZEB1 and E-cadherin upon tetracycline-induced expression (+) of ZEB1-FlagBirA* construct (C termini tag) in the HEK293 Flp-In Trex cell line; all asterisks indicate statistical significance by t-test ($n \geq 3$, *$p \leq 0.05$). (B) Protein expression was assessed by immunoblot of ZEB1, Flag and E-cadherin. Arrows differentiate flagBirA*-tagged ZEB1. (C) 393P cells were pre-transfected with siRNA control or siRNA targeting murine ZEB1 (denoted mZEB1), prior to transfection with FlagBirA*, FlagBirA*-hZEB1 (human ZEB1) or hZEB1-FlagBirA*. Relative mRNA confirms mZEB1 knockdown and hZEB1 overexpression. (D) Transwell assay was conducted to determine whether fusion constructs can functionally replace endogenous ZEB1. (E) Venn diagram depicts comparison of BioID interactors to other AP/MS studies, Zhang et al. 2013 and Gubelman et al. Overlap of the high-confidence BioID ZEB1 interactors identified all known members of the nucleosome remodeling and deacetylase (NuRD; also known as Mi-2) complex as bona fide ZEB1 interacting partners.

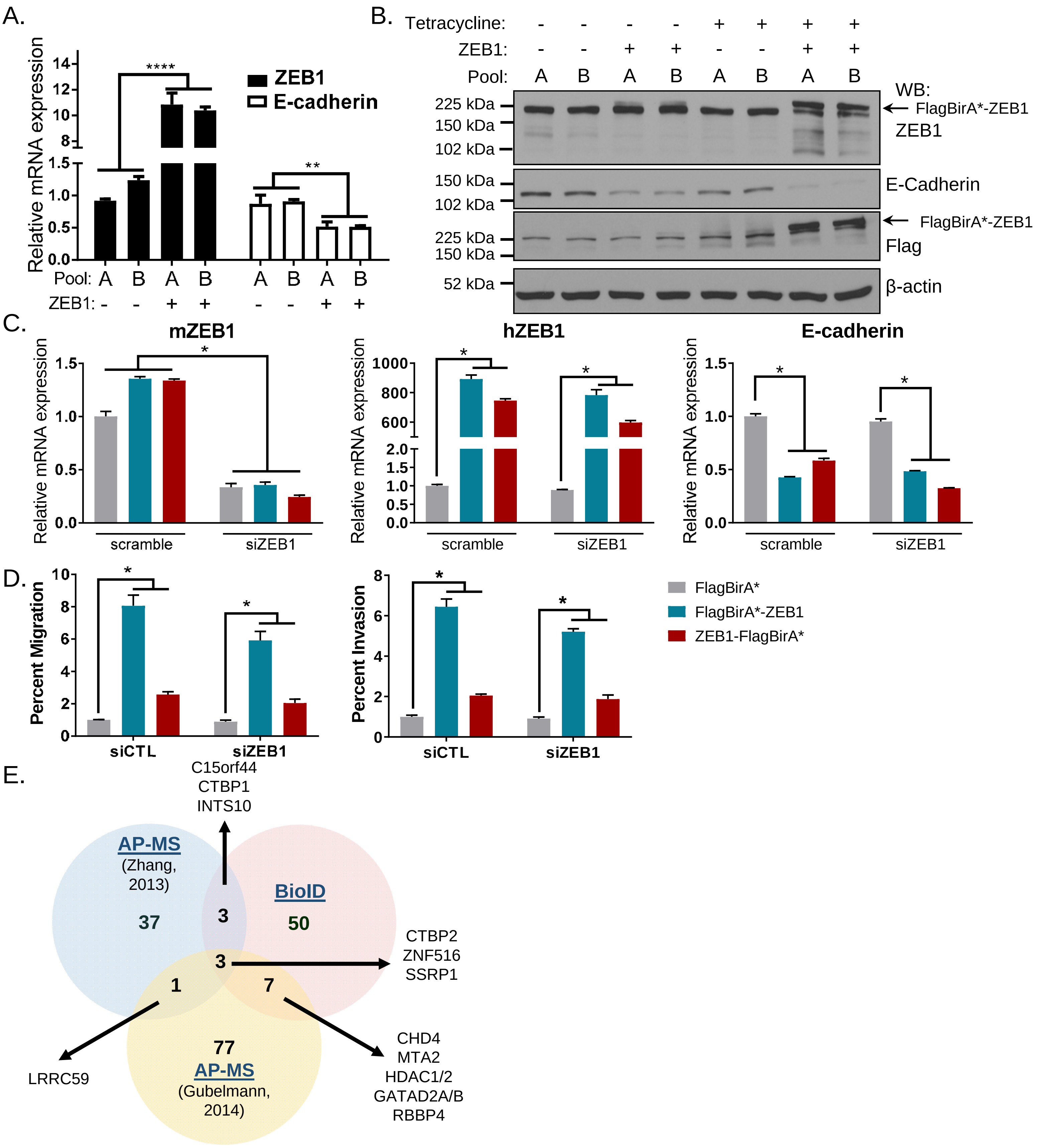

Supplementary Figure 2 (A) Co-immunoprecipitation of MTA2, MTA3, HDAC1, HDAC2, CHD3 or CHD4 suggests no direct association with ZEB1 in the HEK293 Flp-In Trex cell line following 24h FlagBirA*-ZEB1 (Pool A) induction (5% indicates whole cell lysate). Immunoblots demonstrate ability to immunoprecipitate individual components of the NuRD complex. (B) Relative mRNA and protein expression of 344SQ_GFP-ZEB1 cell line over 48h of doxycycline treatment. Cell line exhibits a robust EMT as marked by down regulation of E-cadherin and upregulation of Vimentin and N-Cadherin.

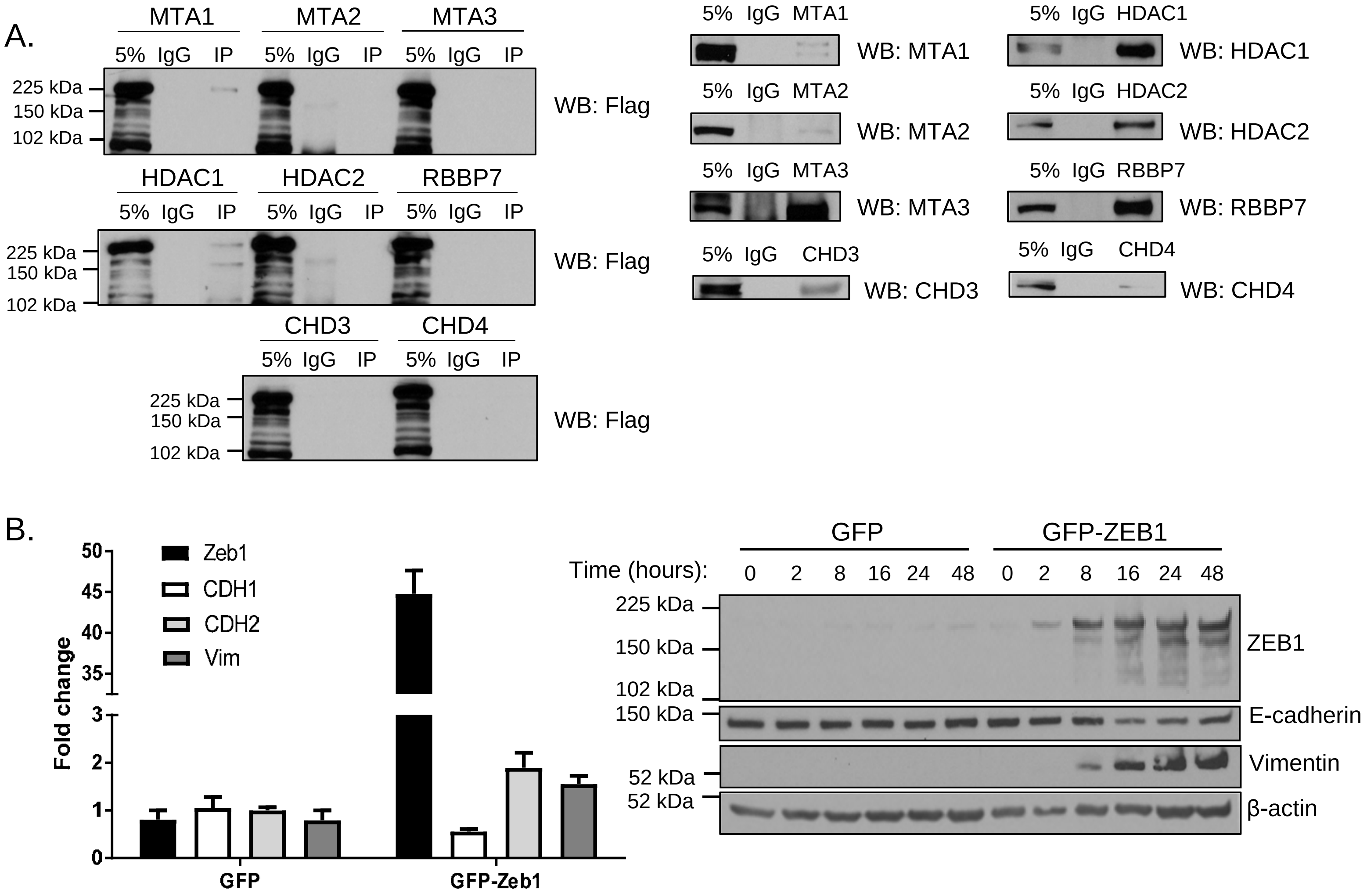

Supplementary Figure 3. Examples of HDAC1-ZEB1, HDAC2-ZEB1 and CHD4-ZEB1 PLA interaction across multiple murine (344SQ and 531LN2) and human (H1299 and H157) lung cancer cell lines. Scale bars represent 200 µm.

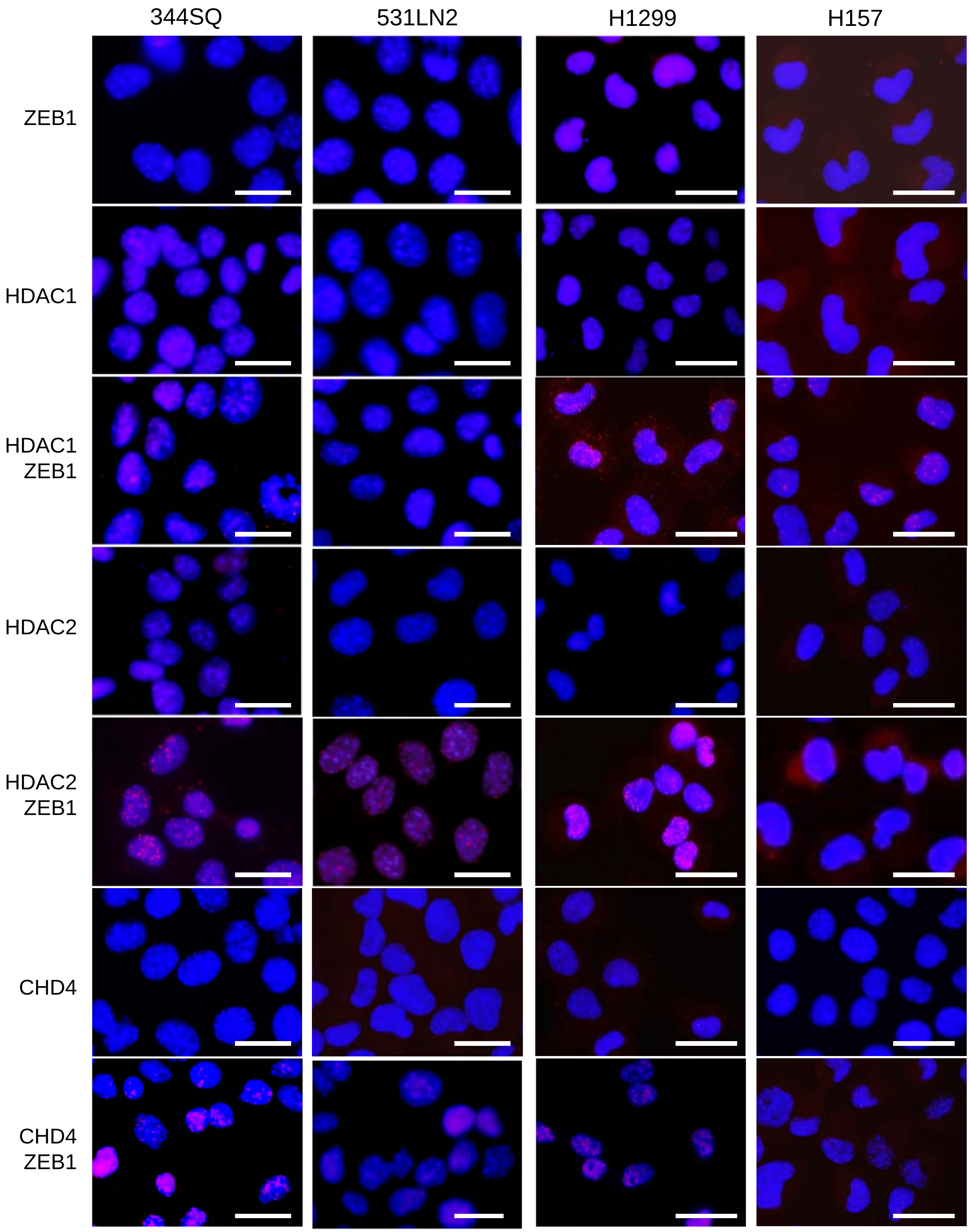

Supplementary Figure 4: (A) Relative mRNA expression of CHD4 after siRNA scramble and CHD4 knockdown in the human NSCLC cell line H1299; all asterisks indicate statistical significance by t-test ($n \geq 3$, *$p \leq 0.05$). (B) Relative ZEB1 binding to the promoters of miR200c-141 in the cell line H1299 after 24h of treatment with the class I HDAC inhibitor, Mocetinostat (1 μM); all asterisks indicate statistical significance by t-test ($n \geq 3$, *$p \leq 0.05$). (C) Immunoblot of ZEB1 and E-cadherin in the cell line H358 after 48h doxycycline induction of GFP vector control or GFP-ZEB1 expression. (D) Relative mRNA expression of ZEB1, CHD4, and miR-200 family members miR200b and miR141 in H358 following pre-transfection of siRNA targeting scramble control or CHD4 and induction of ZEB1 expression for 24h; all asterisks indicate statistical significance by t-test ($n \geq 3$, *$p \leq 0.05$).

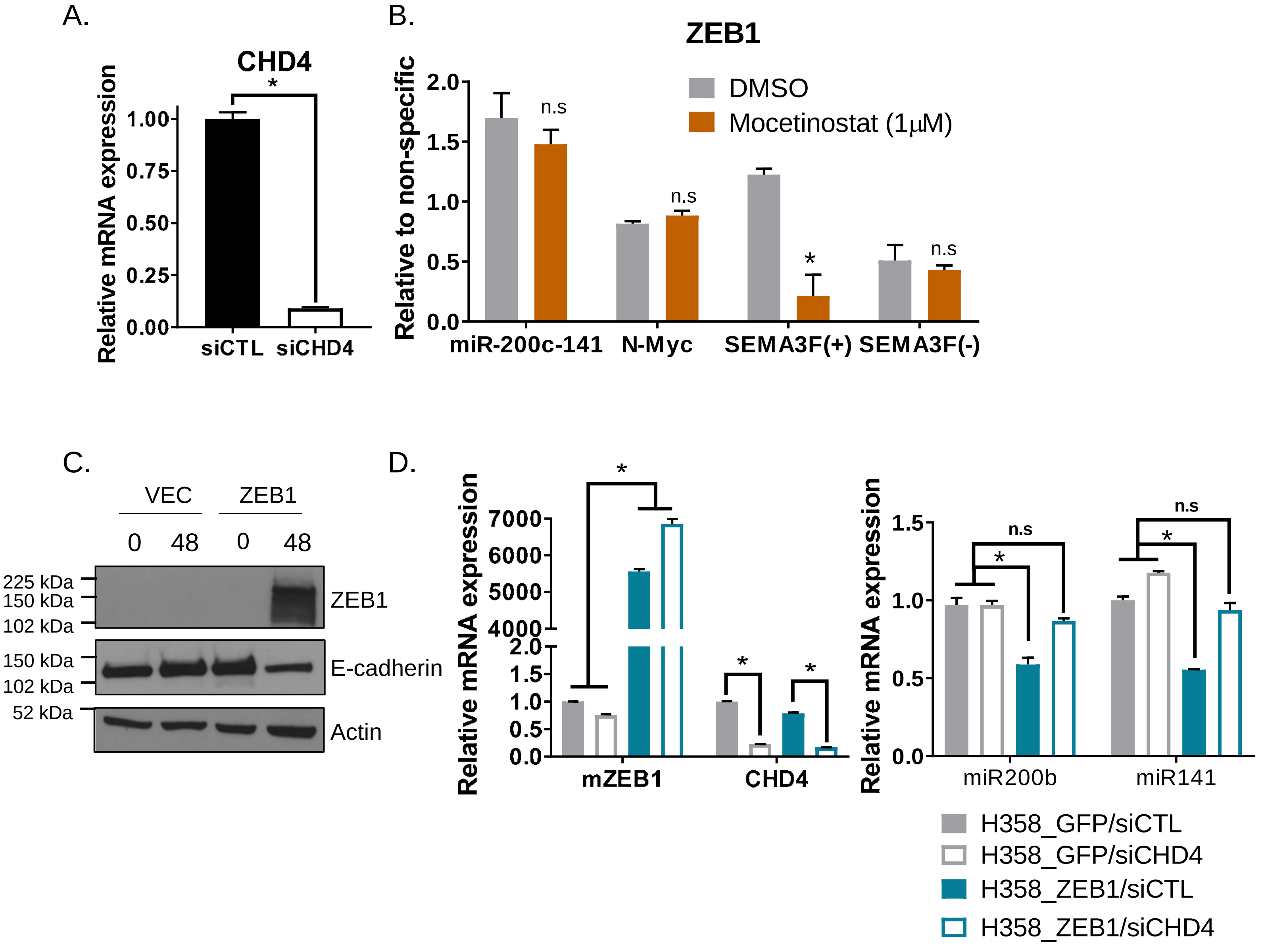

Supplementary Figure 5 (A) Confirmation of ZEB1 and E-cadherin mRNA expression in 393P cell line with constitutive ZEB1 overexpression and (B) 344SQ with 96h of induced miR-200a-b-429 expression; all asterisks indicate statistical significance by t-test ($n \geq 3$, *$p \leq 0.05$). (C) mRNA expression of predicted ZEB1/NuRD targets complex in the cell line 393P with constitutive ZEB1 overexpression; all asterisks indicate statistical significance by t-test ($n \geq 3$, *$p \leq 0.05$). (D) mRNA expression of predicted ZEB1/NuRD targets complex in the cell line 344SQ with 96h of induced miR-200a-b-429 overexpression; all asterisks indicate statistical significance by t-test ($n \geq 3$, *$p \leq 0.05$). (E) Relative ZEB1 binding in the cell line H1299 following CHD4 knockdown; all asterisks indicate statistical significance by t-test ($n \geq 3$, *$p \leq 0.05$). (F) Relative ZEB1 binding to the promoters of TBC1D2a, TBC1D2b, and EPS8L2 in the cell line H1299 following 24 h of treatment with the class I HDAC inhibitor, Mocetinostat (1 μM); all asterisks indicate statistical significance by t-test ($n \geq 3$, *$p \leq 0.05$).

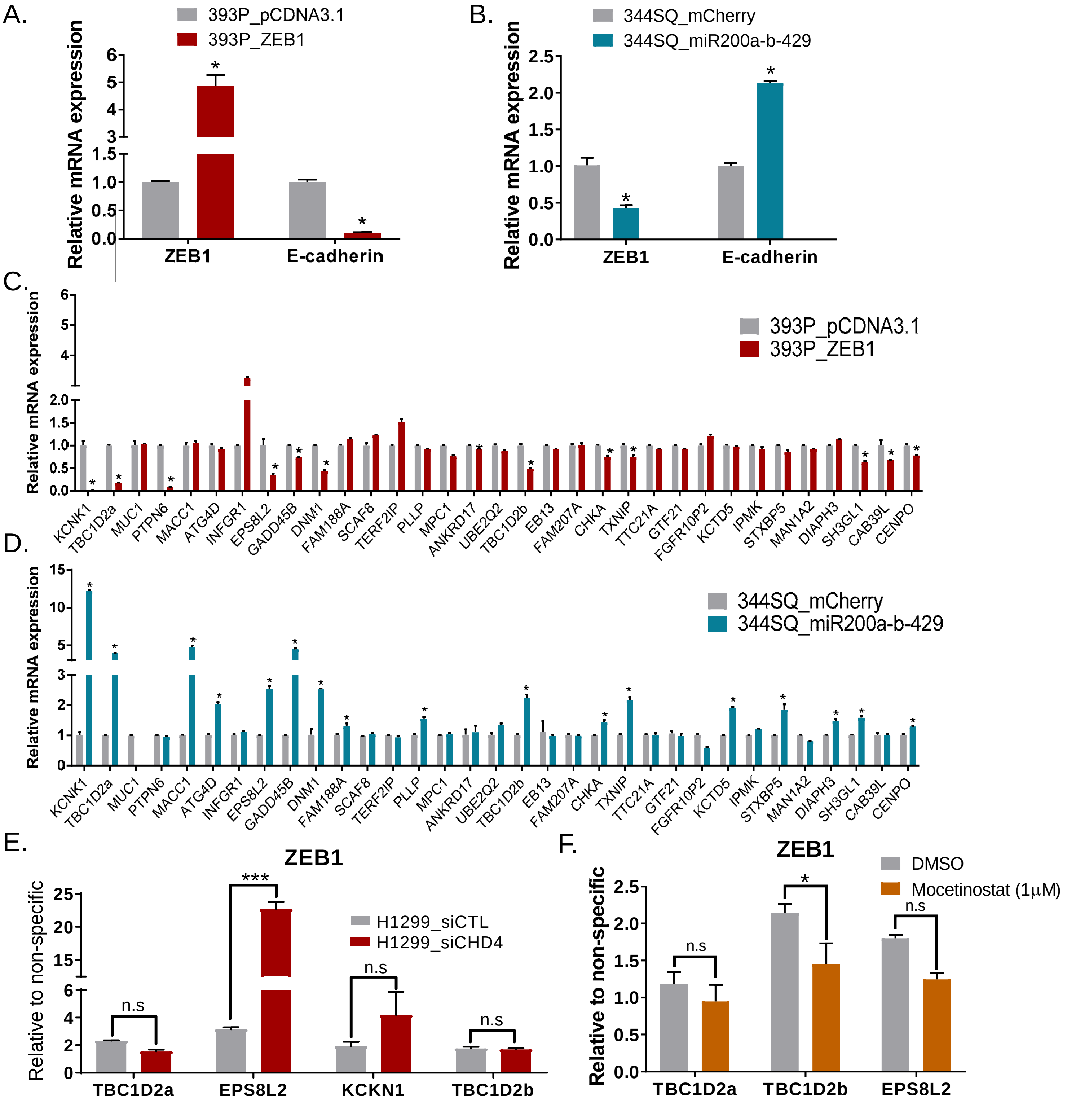

Supplementary Figure 6 (A) TBC1D2a, TBC1D2b, and EPS8L2 were transiently expressed in the cell line 344SQ for 24h and compared to pLenti empty vector control. Relative mRNA for each gene was confirmed by qRT-PCR; all asterisks indicate statistical significance by t-test ($n \geq 3$, *$p \leq 0.05$). (B) Immunoblot for TBC1D2a, TBC1D2b, EPS8L2 and E-cadherin. (C) Boyden chamber migration and invasion assays were performed over 16h, after 24h of transient transfection of each gene; all asterisks indicate statistical significance by t-test ($n \geq 3$, *$p \leq 0.05$).

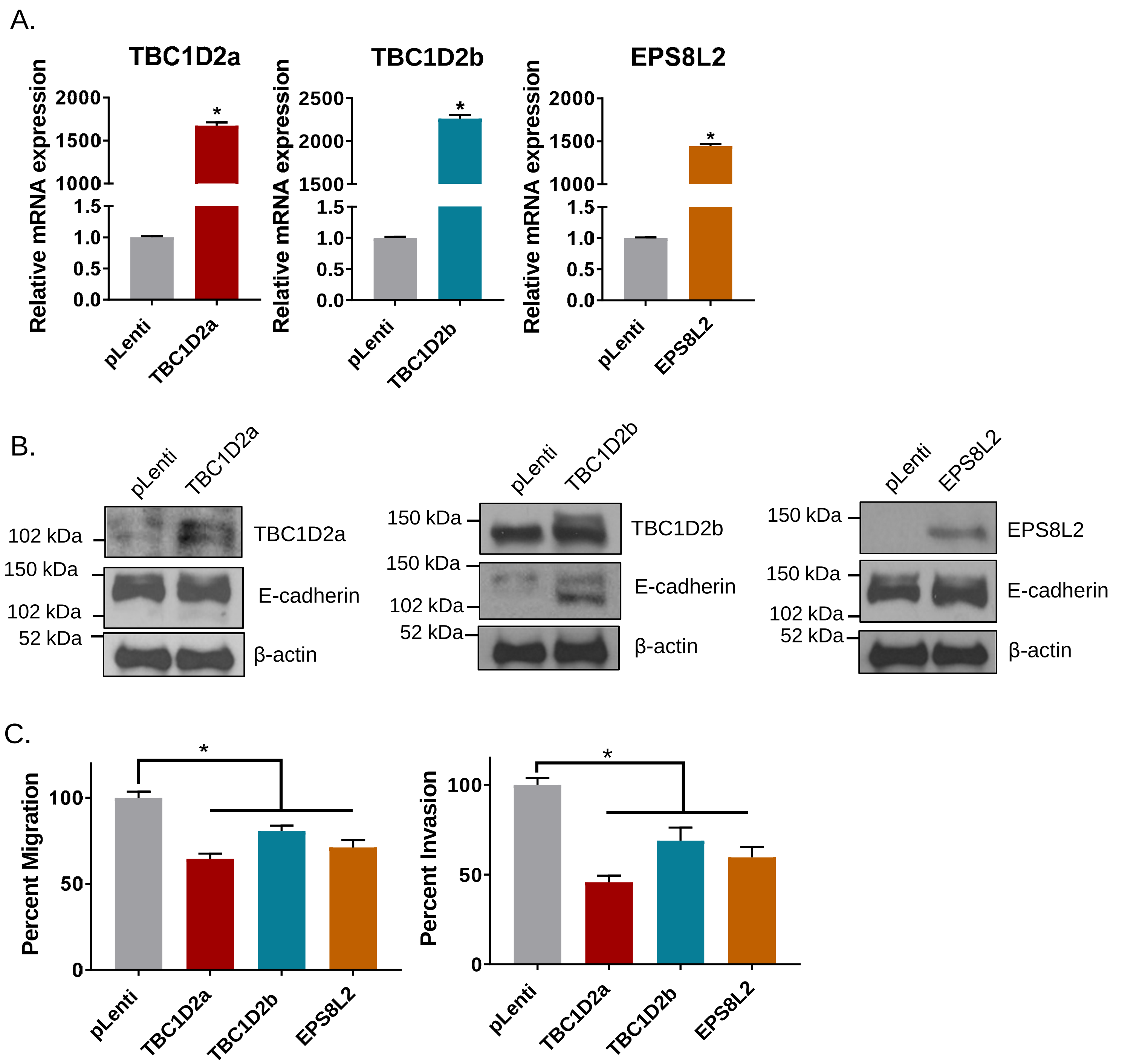

Supplementary Figure 7 (A) Relative TBC1D2b mRNA expression in murine cell lines 344SQ and 531LN2 upon doxycycline induction over a 48h time-course. (B) Representative immunofluorescence images of TBC1D2b (green) and counterstain DAPI (blue) in 344SQ cell line following 24h of doxycycline induction. Scale bars represent 50 µm. (C) Quantification (left) of wound closure assay (right) was conducted following 24h of TBC1D2b overexpression; all asterisks indicate statistical significance by t-test (n ≥5, *p ≤ 0.0005). (D) Immunoblot and relative mRNA expression of TBC1D2b upon stable shRNA knockdown in 344SQ_shTBC1D2b cell lines with multiple independent shRNAs (sh1-sh4) vs a non-targeting scramble control (scr). (E) Transwell migration/invasion assays after downregulation of TBC1D2b in the murine cell lines 344SQ_shTBC1D2b (sh1-sh4). (F) Relative mRNA expression of Rab22 in cell lines 393P and H358 following exogenous overexpression of the human GFP-Rab22 over a 24h time-course.

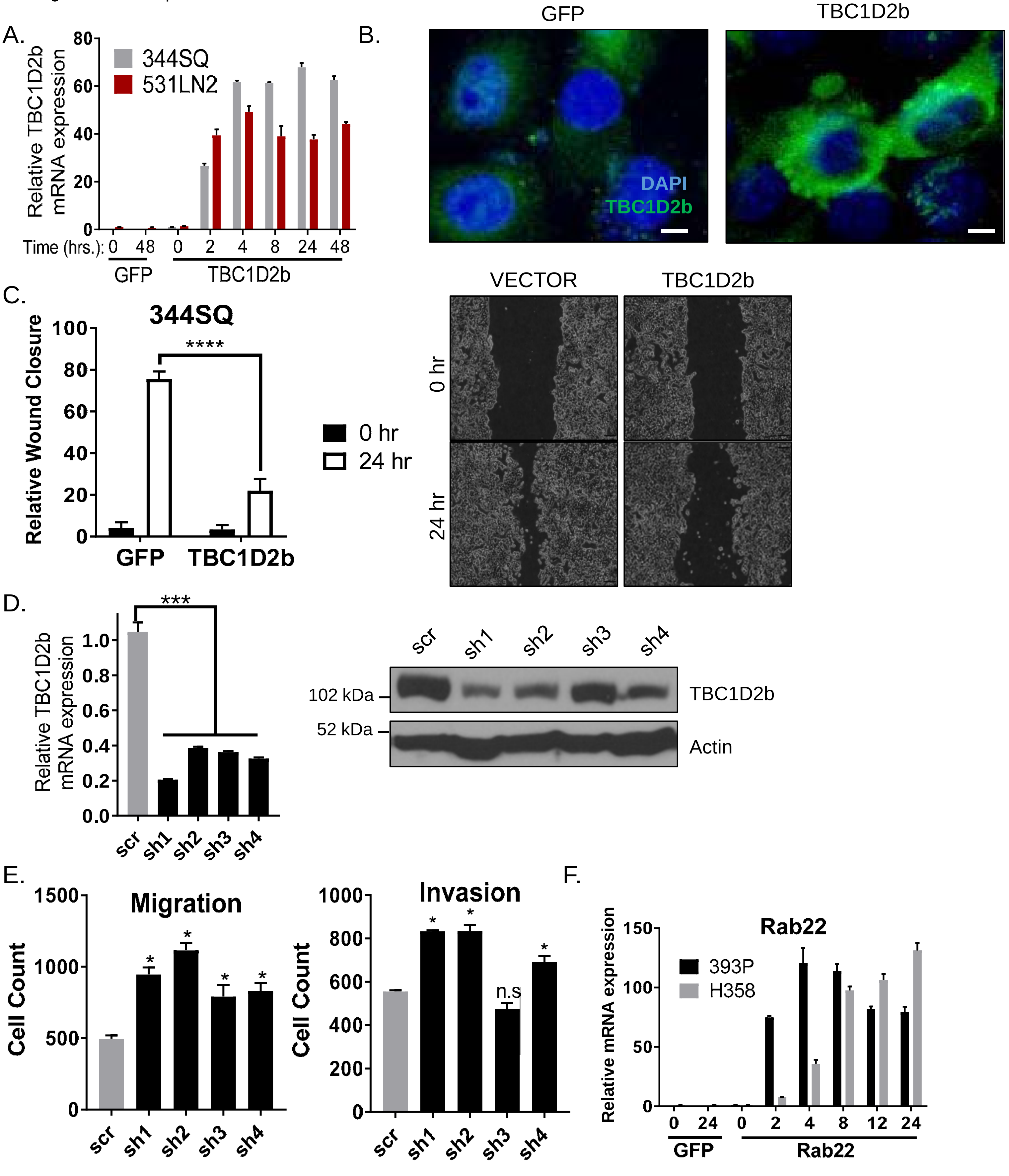

Supplementary Figure 8 (A) Relative E-cadherin mRNA expression in murine cell lines 344SQ and 531LN2 demonstrates that E-cadherin transcription is not impacted by induced TBC1D2b expression until ~24 h of overexpression; standard deviation n=3; *p<0.05. (B) Relative mRNA expression and immunoblot of E-cadherin upon constitutive knockdown of TBC1D2b in 344SQ cell lines; all asterisks indicate statistical significance by t-test ($n \geq 3$, *$p \leq 0.05$). (C) Immunoblot of E-cadherin in H358 cells expressing human GFP-tagged Rab22. (D) 344SQ-TBC1D2b (induced for 24h) lysate was untreated (-) or treated with lambda phosphatase (+) for 30 min. in the presence of protease inhibitors and then blotted for E-cadherin or total p-Tyrosine. Arrows indicate the differential observed in the molecular weight of E-cadherin following treatment.

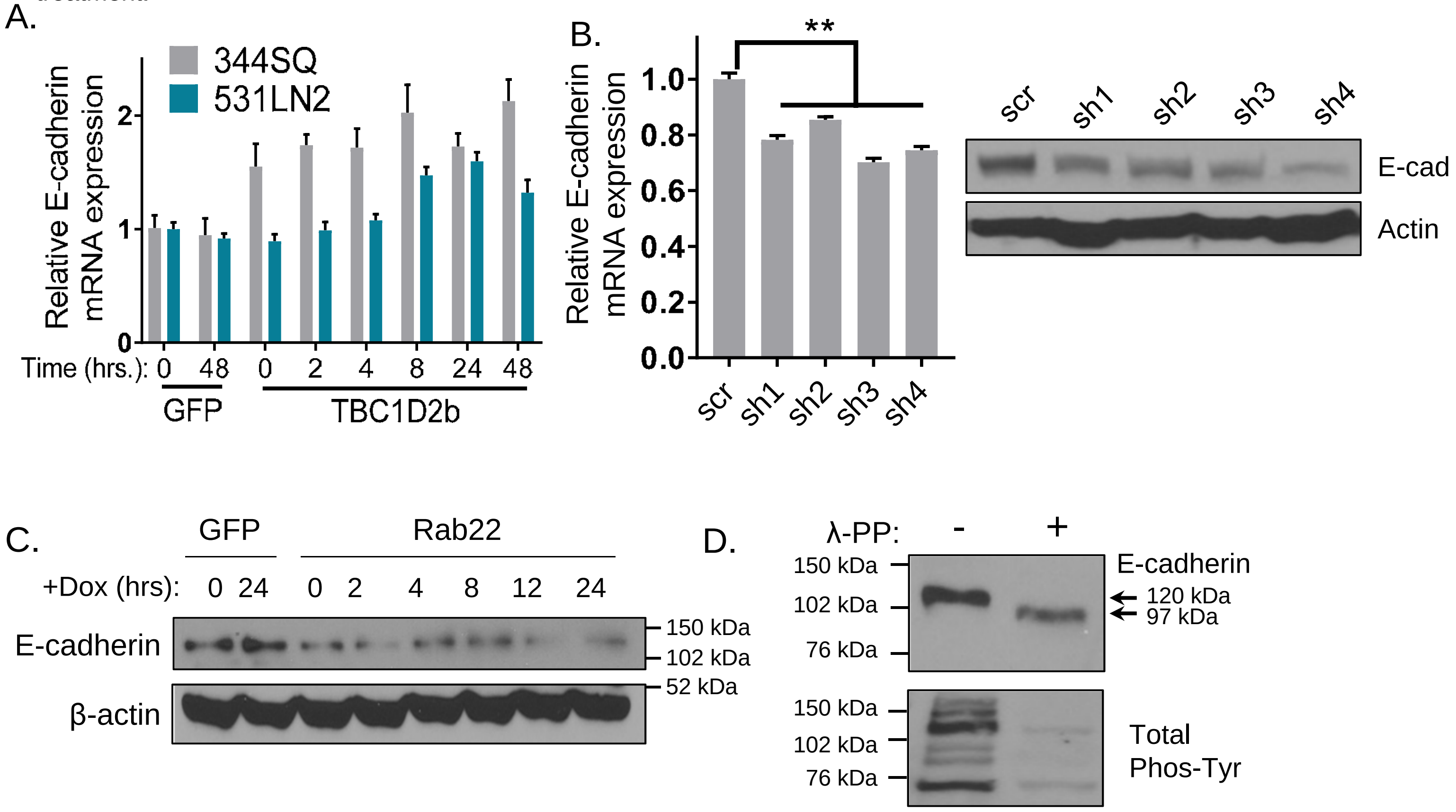

Supplementary Figure 9 (A) Relative mRNA expression of TBC1D2b and E-cadherin in 344SQ_GFP or 344SQ_TBC1D2b tumors confirms overexpression. Cell lines were implanted for one week prior to induction with doxycycline feed; all asterisks indicate statistical significance by t-test (n ≥ 3, *p ≤ 0.05). (B) Relative mRNA expression of 344SQ tumors expressing PLKO scramble control or TBC1D2b knockdown shRNA2 display both reduced TBC1D2b and E-cadherin expression; all asterisks indicate statistical significance by t-test (n ≥ 3, *p ≤ 0.05).

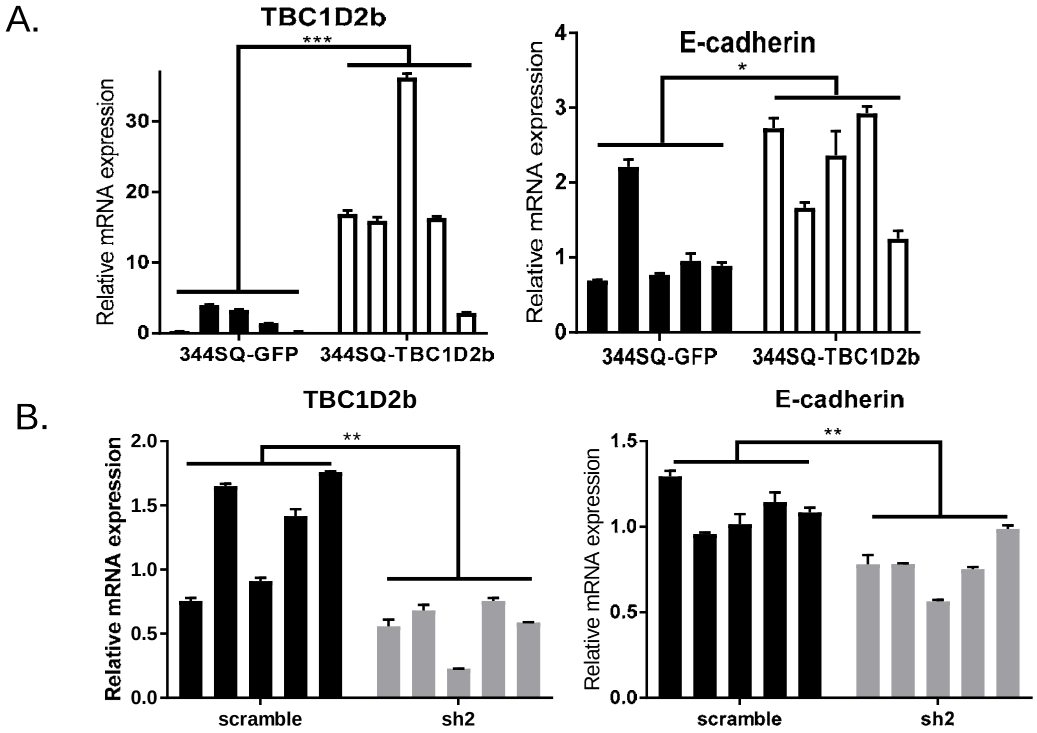

Supplementary Figure 10: Uncropped blots for Figure 1A.

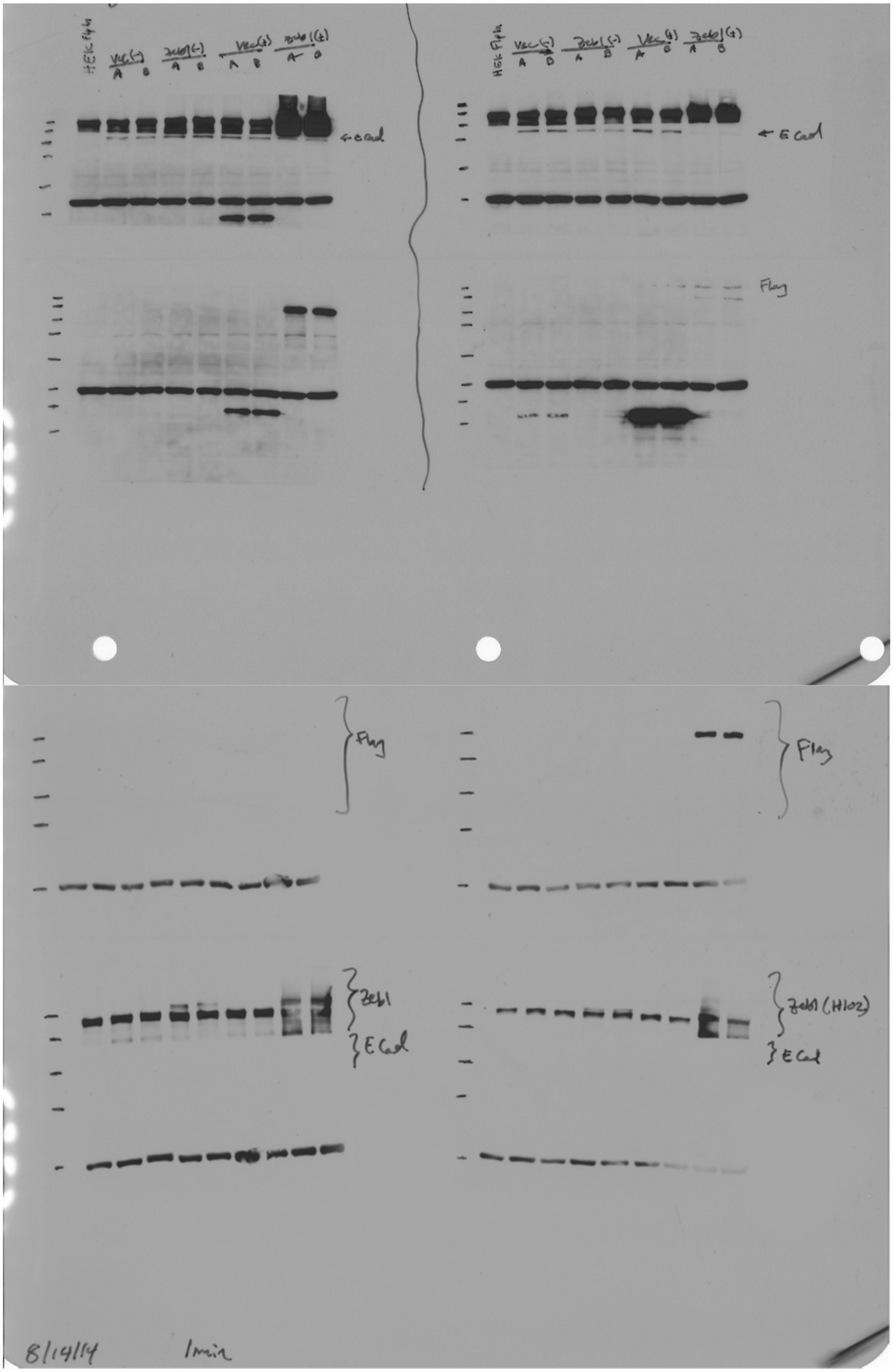

Supplementary Figure 10: Uncropped blots for Figure 2A.

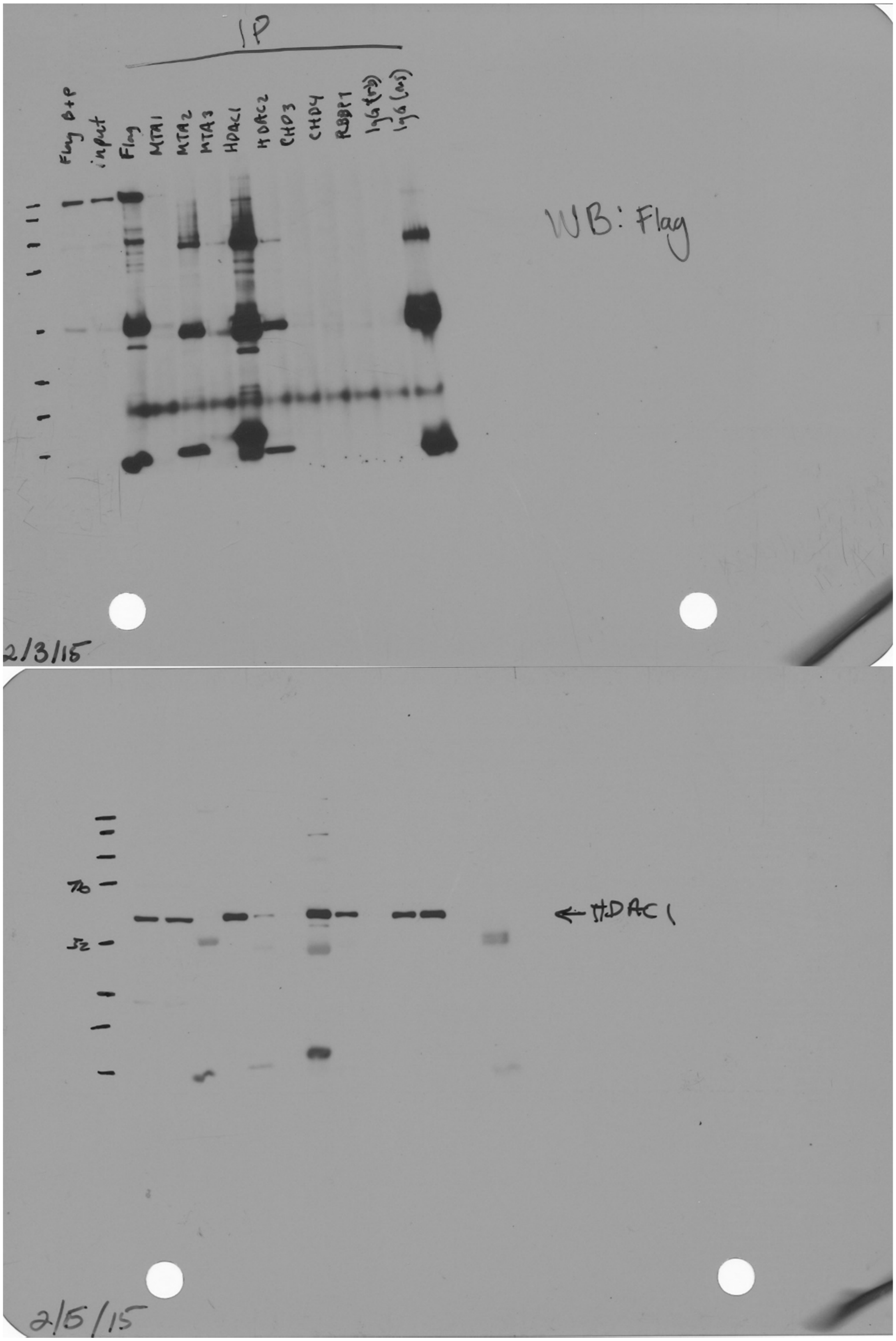

Supplementary Figure 10: Uncropped blots for Figure 2B.

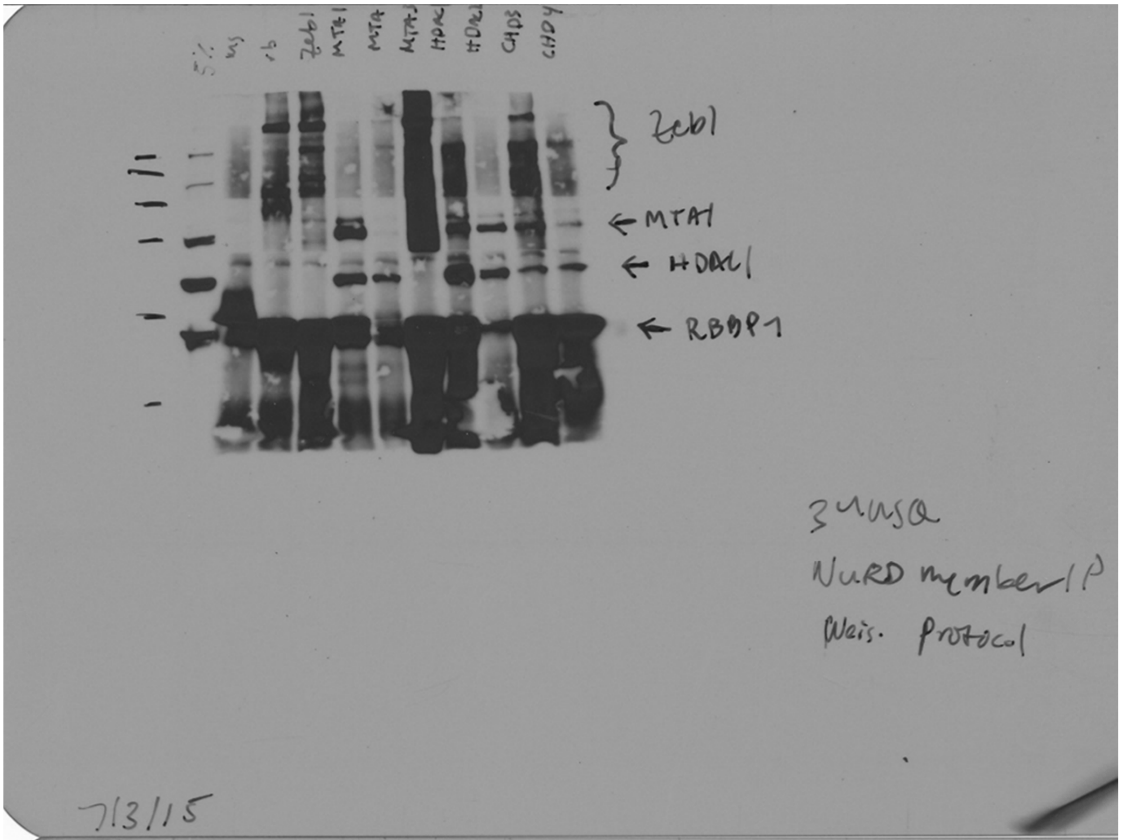

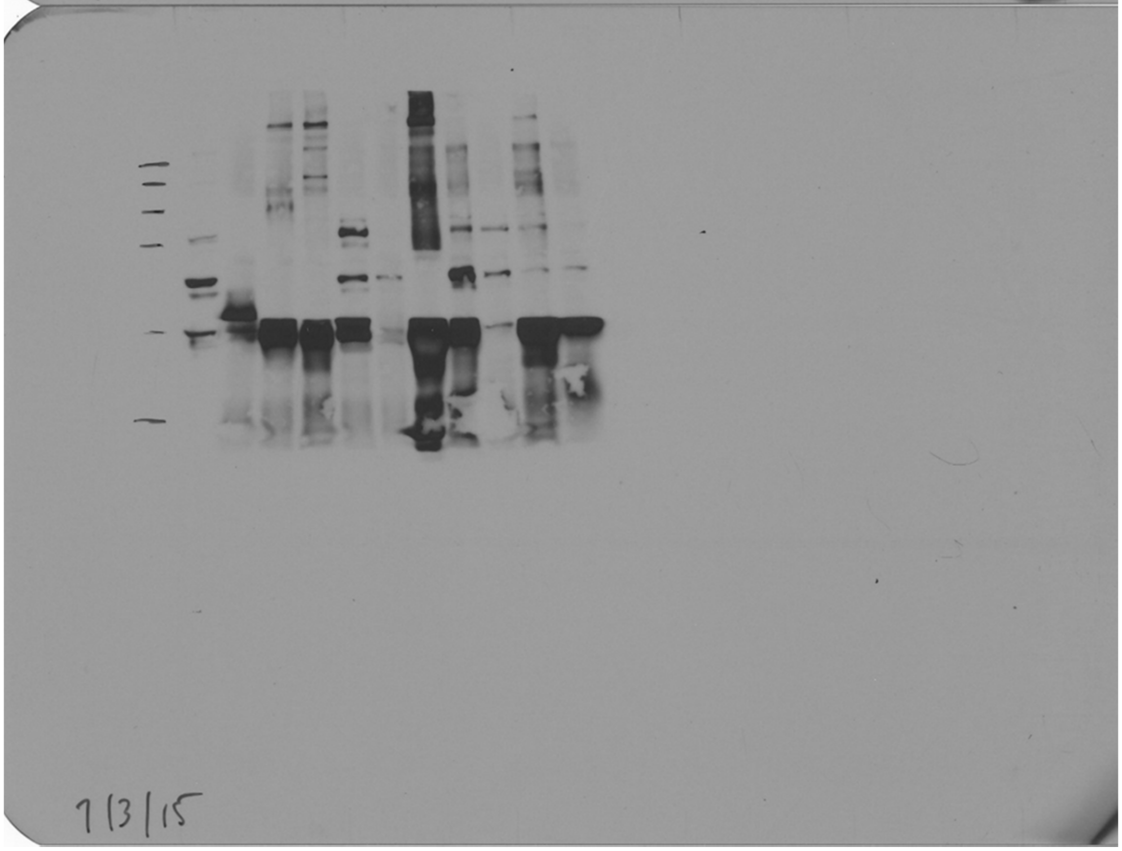

Supplementary Figure 10: Uncropped blots for Figure 2E.

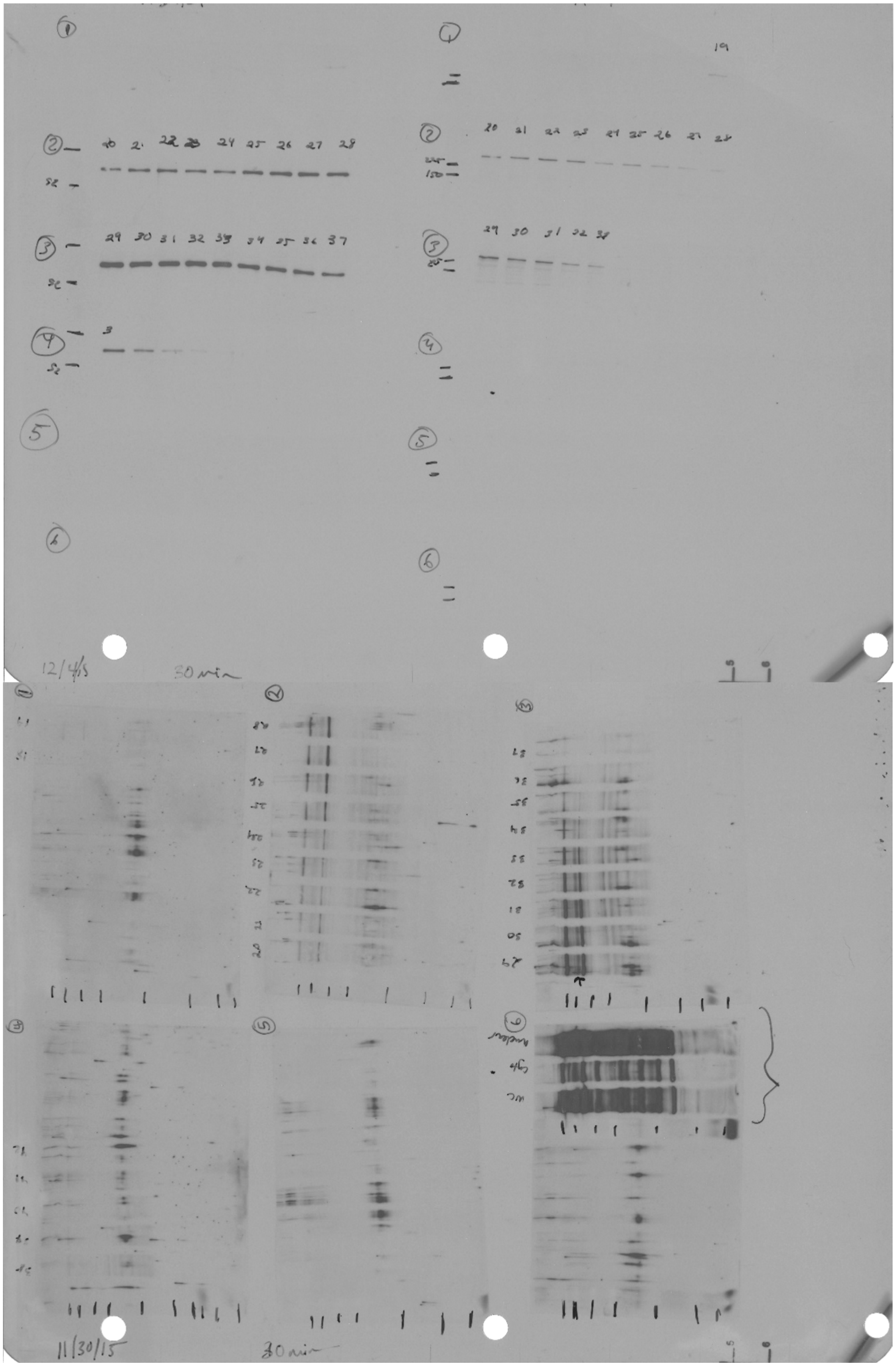

Supplementary Figure 10: Uncropped blots for Figure 2F.

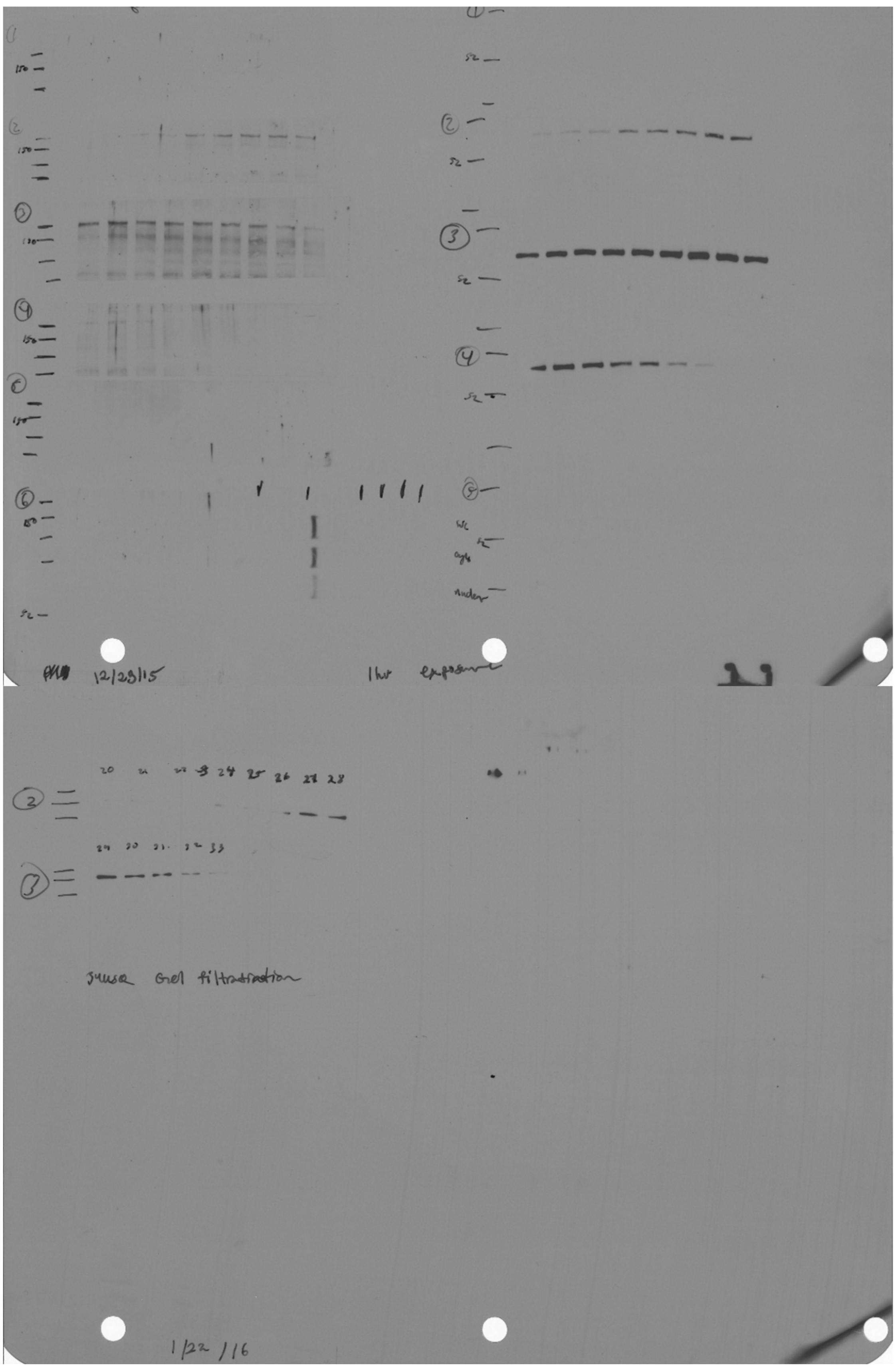

Supplementary Figure 10: Uncropped blots for Figure 5A.

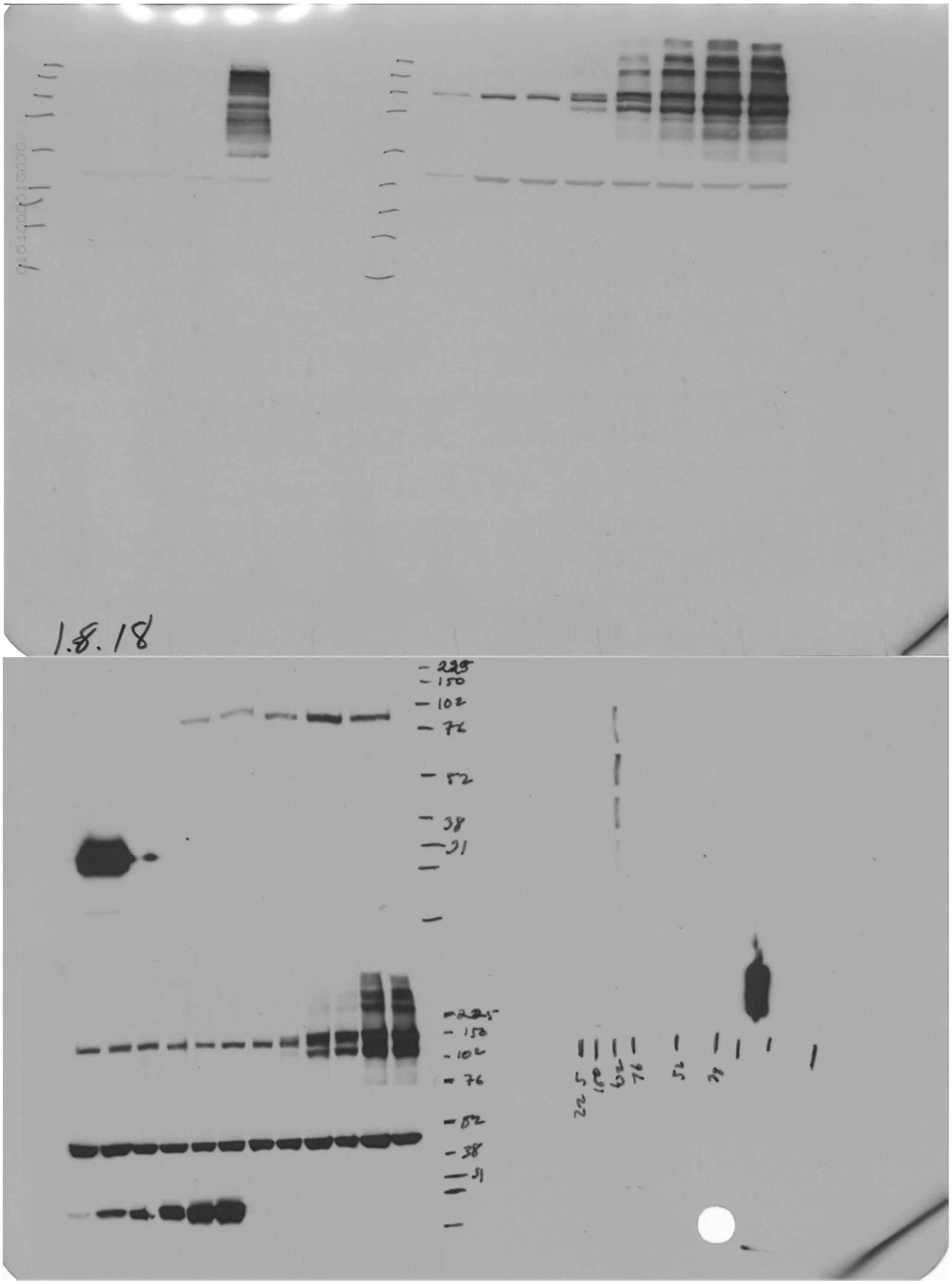

Supplementary Figure 10: Uncropped blots for Figure 5B.

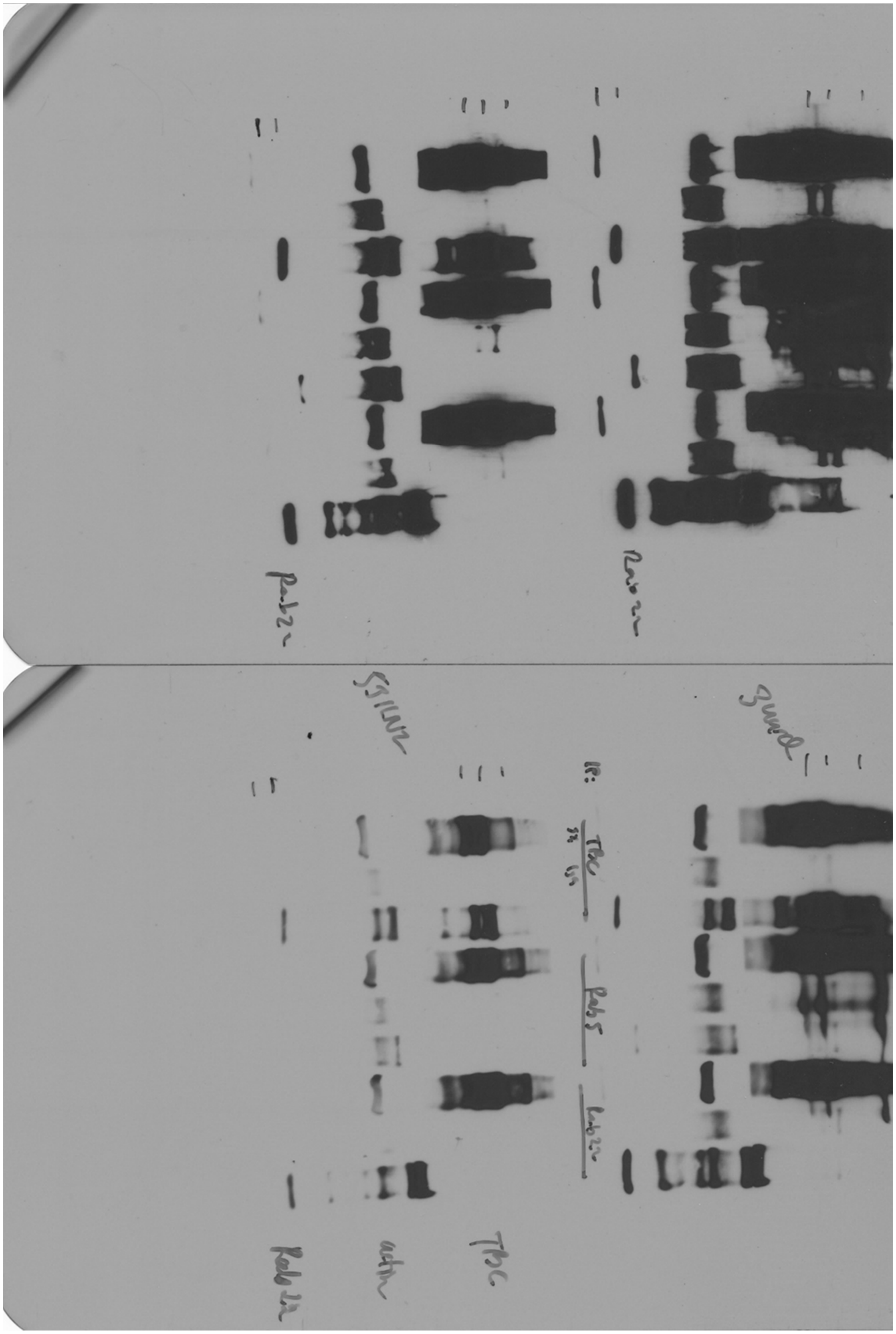

Supplementary Figure 10: Uncropped blots for Figure 5D.

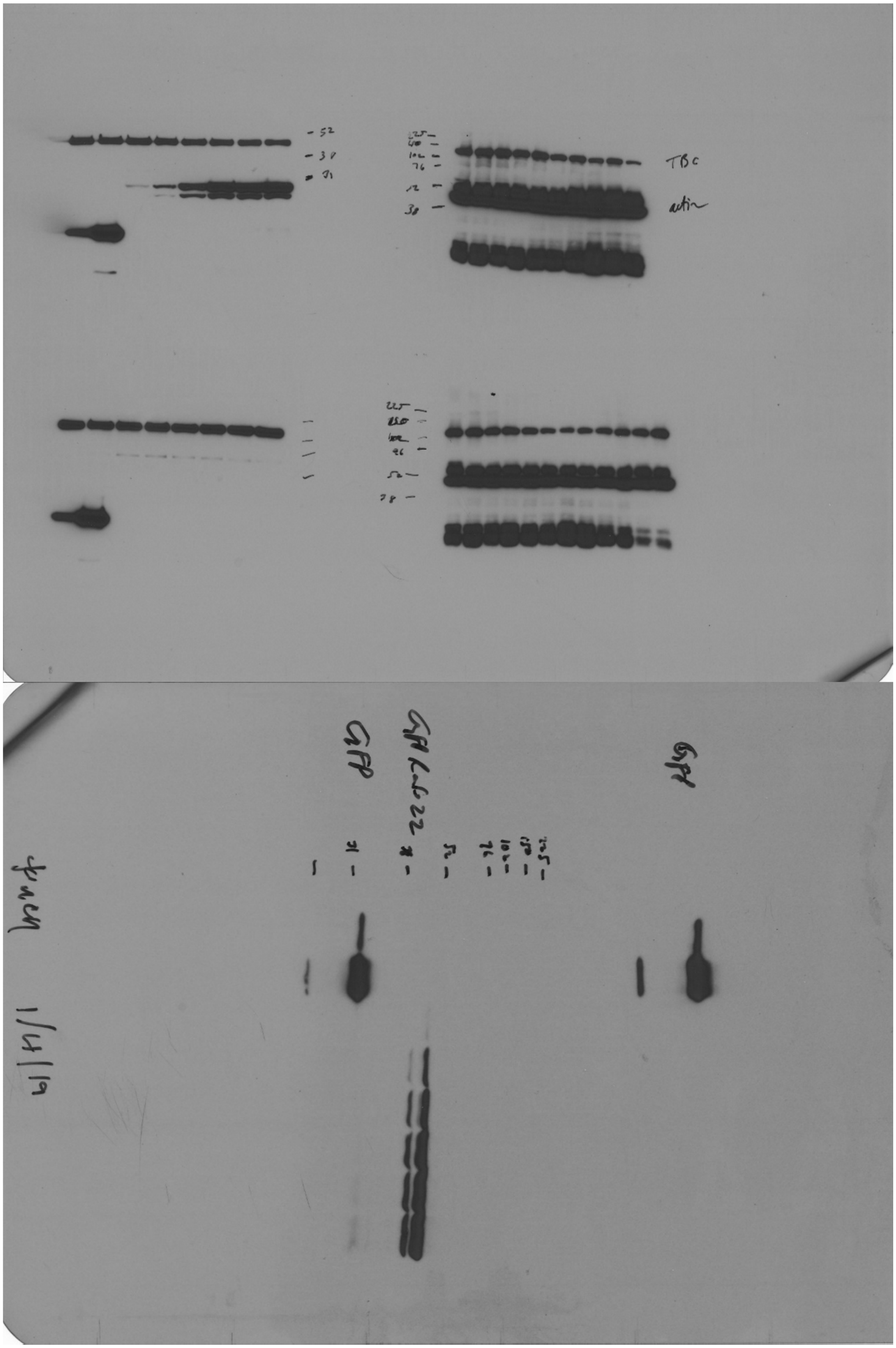

Supplementary Figure 10: Uncropped blots for Figure 6A.

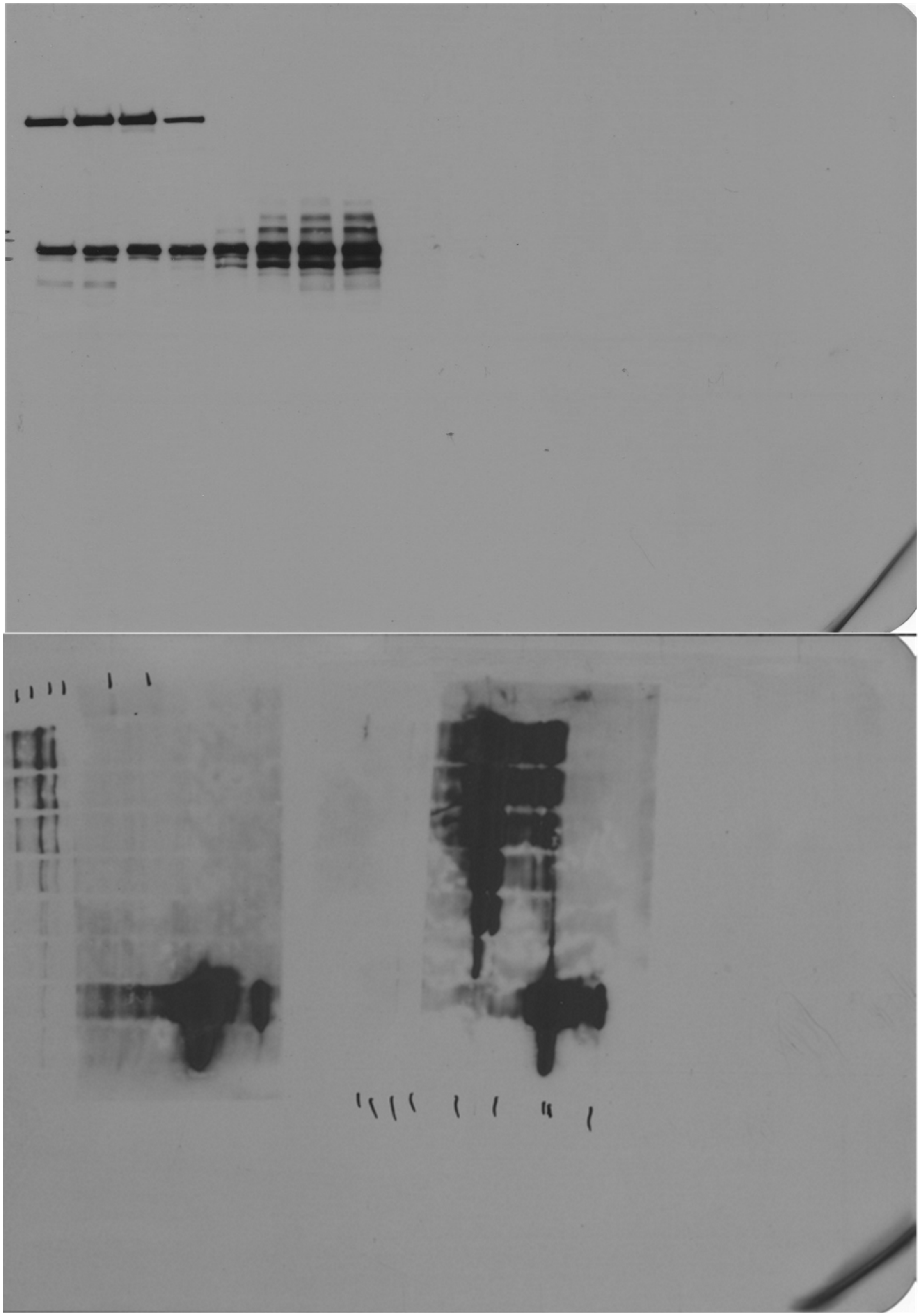

Supplementary Figure 10: Uncropped blots for Figure 6B.

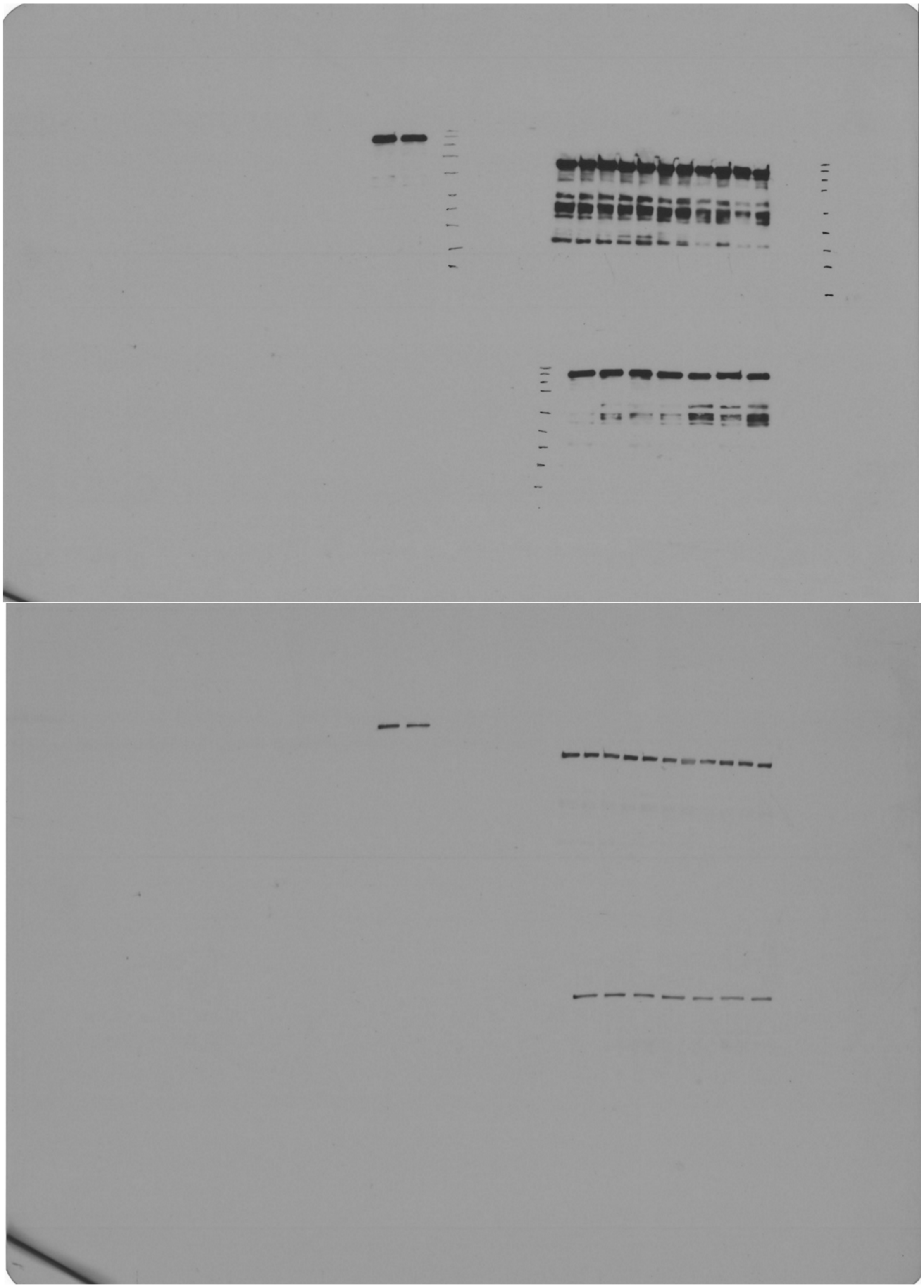

Supplementary Figure 10: Uncropped blots for Figure 6C.

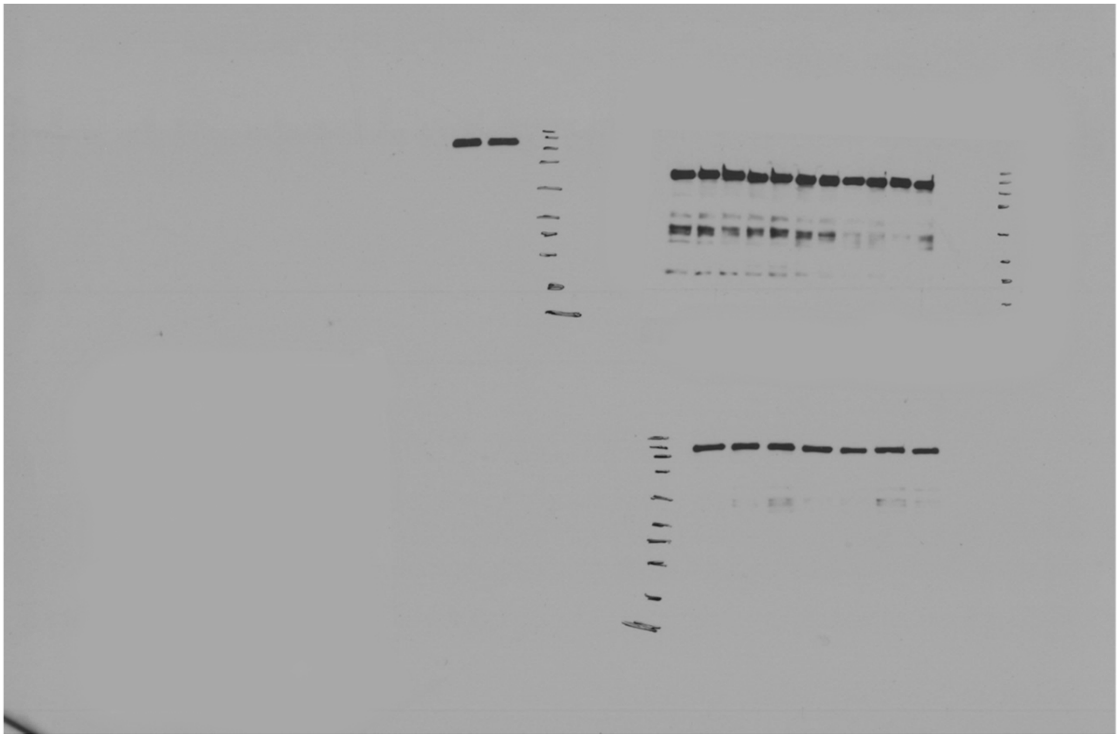

Supplementary Figure 10: Uncropped blots for Figure 6E.

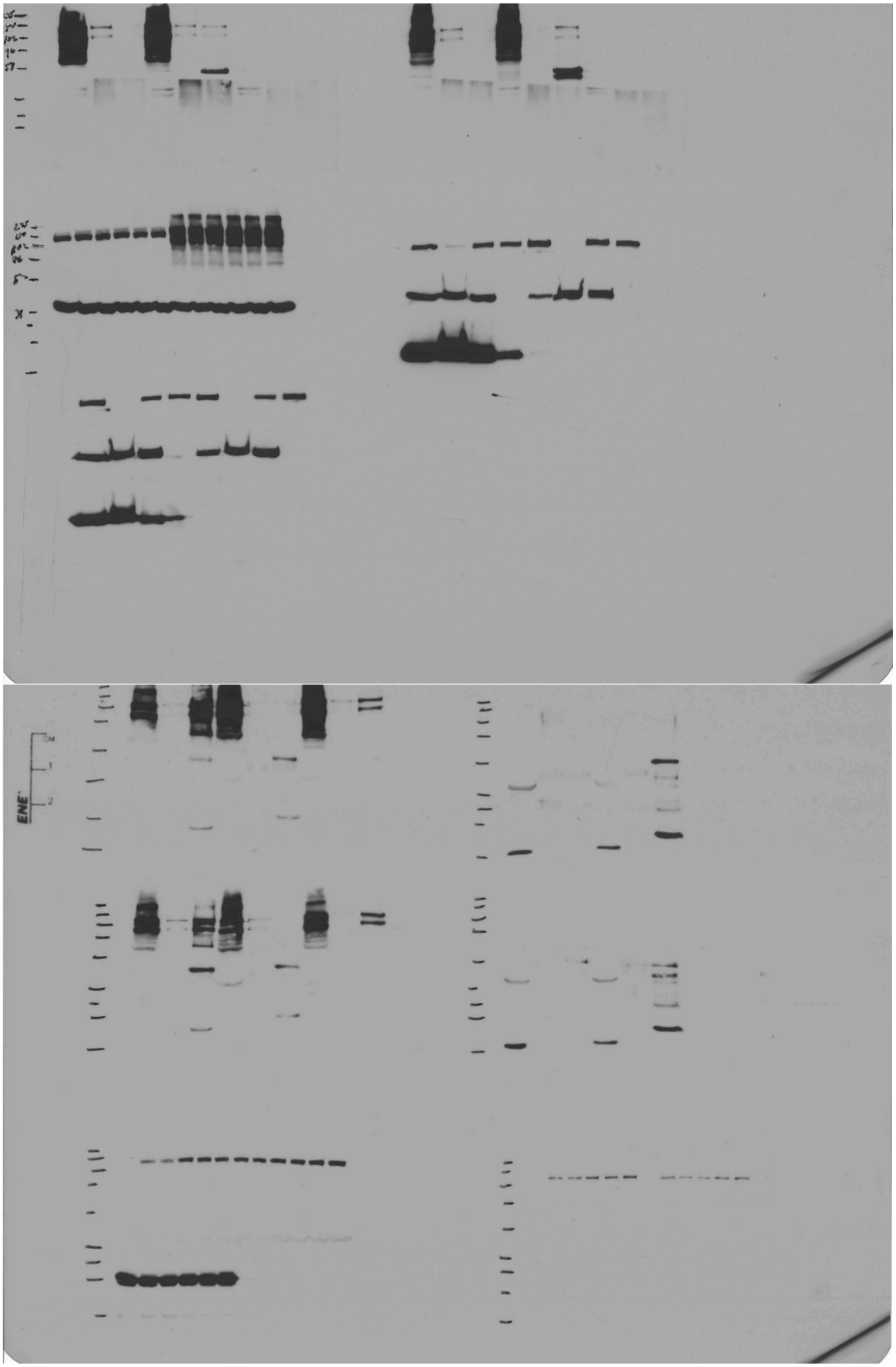

Supplementary Figure 10: Uncropped blots for Figure 6F.

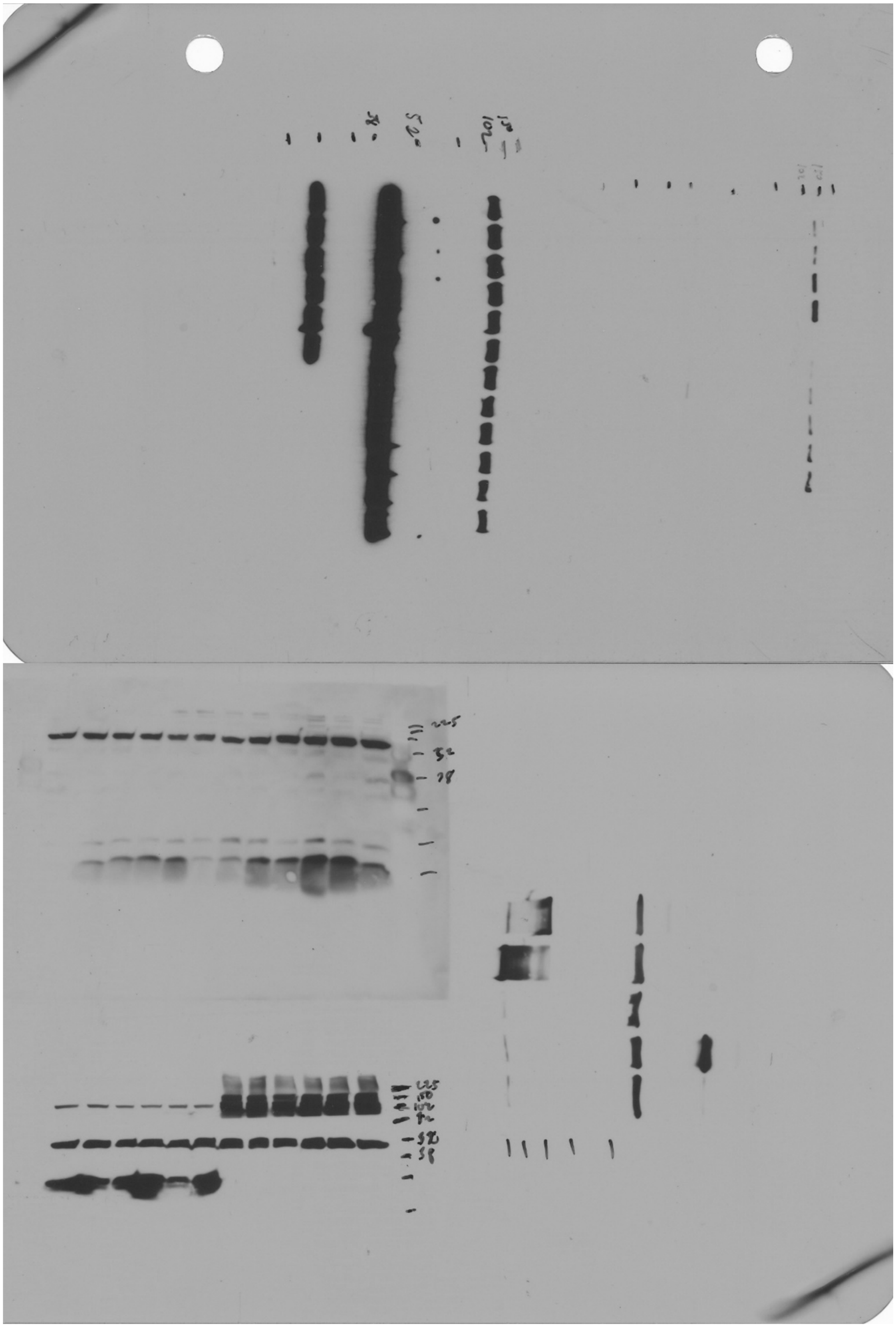

Supplementary Figure 10: Uncropped blots for Figure 7B.

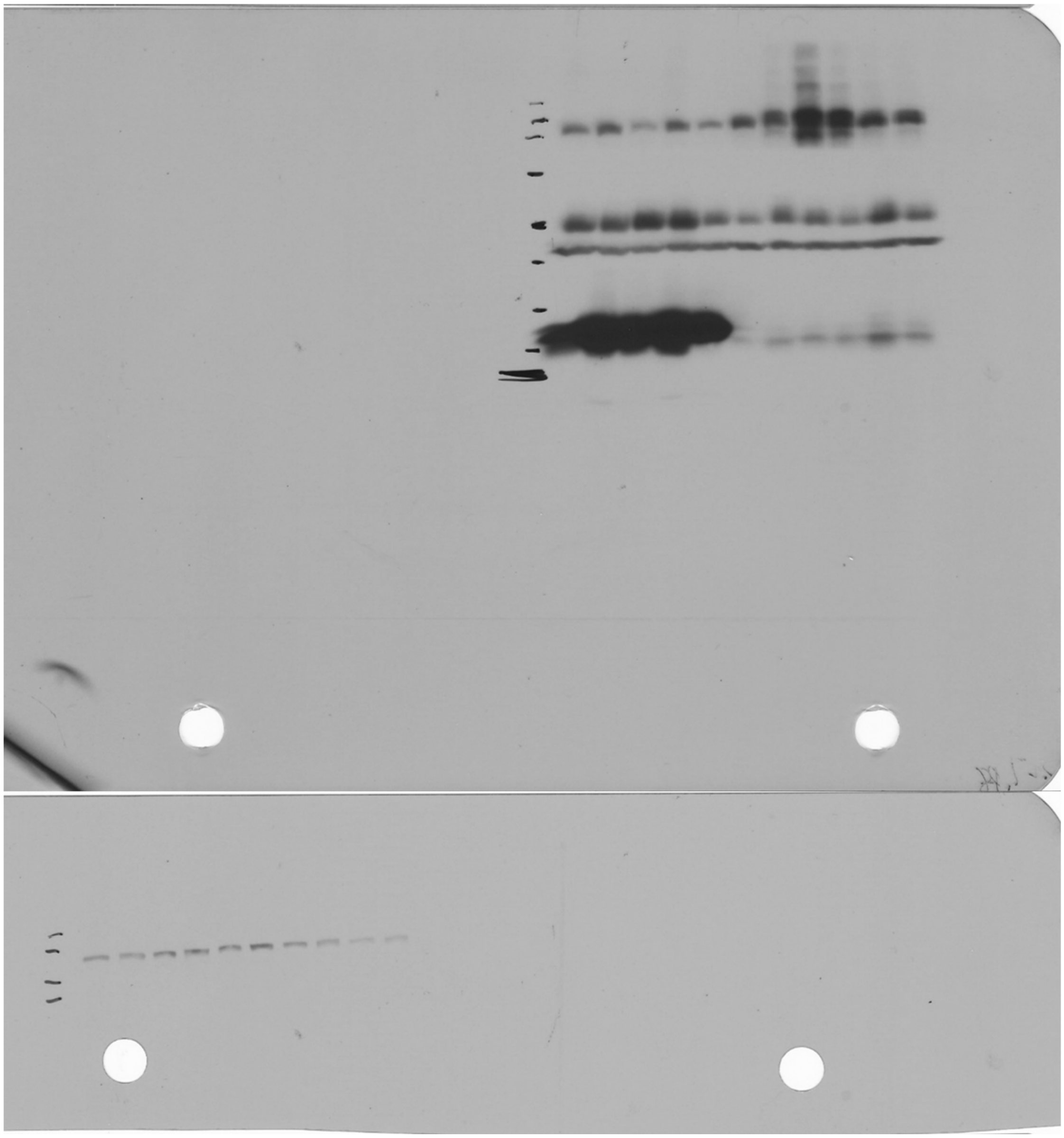

Supplementary Figure 10: Uncropped blots for Figure 7E.

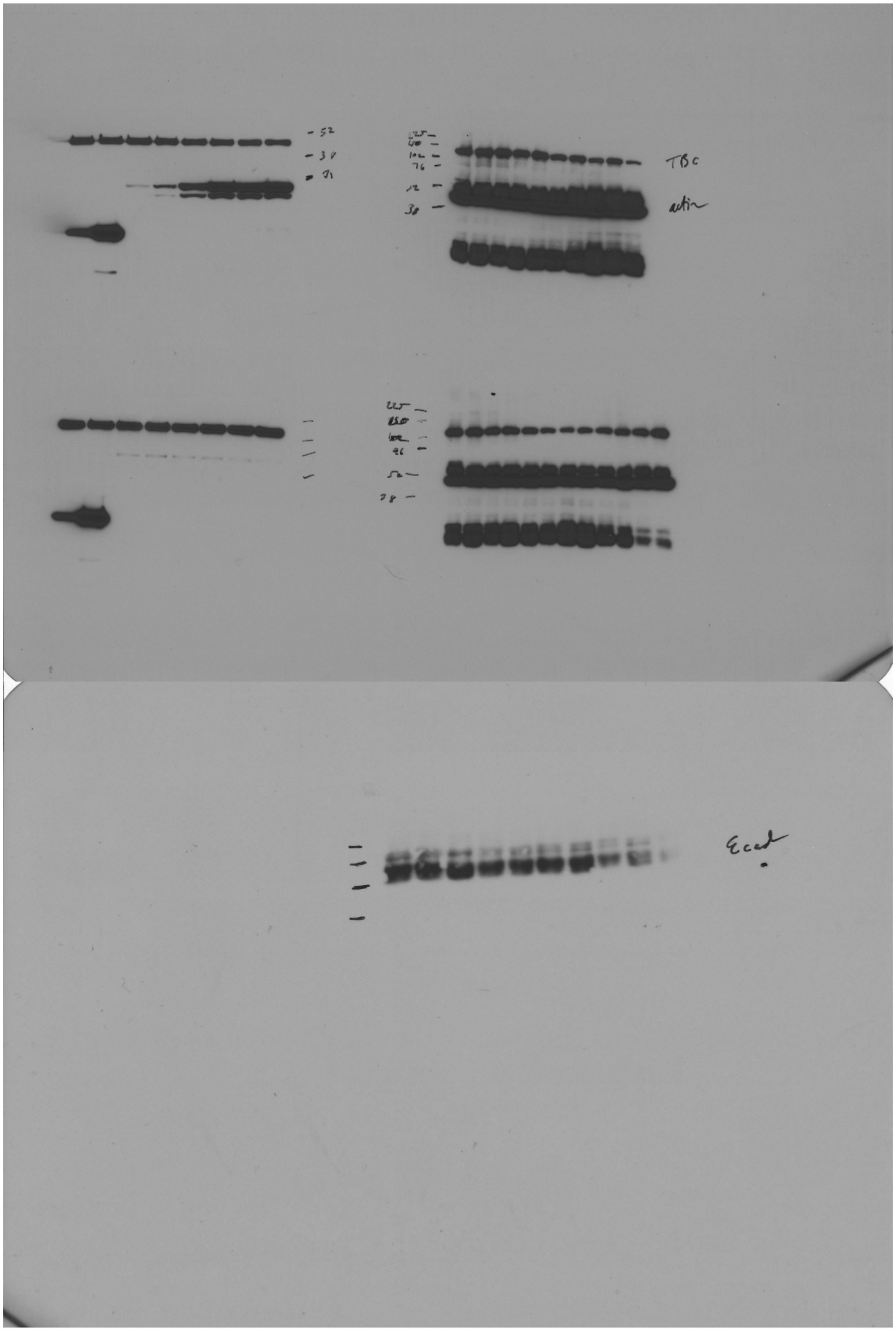

Supplementary Table 1: qPCR primer sequences

Gene	qPCR Primer Sequence
KCNK1-F (ms)	TTCCAGGGGAAGGCTACAACCA
KCNK1-R (ms)	CTCGTGGAGTTCACAGAAGGTC
TBC1D2-F (ms)	CAGGAGAGGATGGAGCATCTGA
TBC1D2-R (ms)	ACTTGGTCAGCAGGGCTTTCTC
MUC1-F (ms)	AGTGCCTCTGACGTGAAGTCAC
MUC1-R (ms)	GGGAGGGAACTGCATCTCATTC
PTPN6-F (ms)	TTGGCAGGAGAACACTCGTGTC
PTPN6-R (ms)	TGCTCCCTACTGTTGGTCACAG
MACC1-F (ms)	TATTACAGCACGGCAAGGGC
MACC1-R (ms)	TTAGCTGCGTGATGTCCTCC
ATG4D-F (ms)	GTCTACATCGGCTAGTGGAGCT
ATG4D-R (ms)	GACTTCTGAGCAACTCTCCACAG
IFNGR1-F (ms)	CTTGAACCCTGTCGTATGCTGG
IFNGR1-R (ms)	TTGGTGCAGGAATCAGTCCAGG
EPS8L2-F (ms)	AGCCAGACAGTGCTAAACCAGC
EPS8L2-R (ms)	GAGCACTCTCAATGTCTTCCTGC
GADD45B-F (ms)	GGAGACATTGGGCACAACCGAA
GADD45B-R (ms)	CTGCTCTCTTCACAGTAACTGGC
DNM1-F (ms)	GTGGACATGGTTATCTCGGAGC
DNM1-R (ms)	GGTGGTCACAATTCGCTCCATC
FAM188A-F (ms)	CTCGGTATCCATGAACAAGCAGC
FAM188A-R (ms)	GTGAGTTTCGCTGCCAACAATCC
GTPBP2-F (ms)	GGACTGTGGTTGGAGGAACACT
GTPBP2-R (ms)	ACCTGCTCGAAGAACACGACAC
TERF2IP-F (ms)	GAGAACTCCAGATTTGCCTGAAG
TERF2IP-R (ms)	AATCAGGAGGGCTCTCATCCAC
SCAF8-F (ms)	AGACCTTCAACAGCGAGTTGTAT
SCAF8-R (ms)	CTTAATAGCTTTGATGGCTGCCT
PLLP-F (ms)	GTTTGTCGCTGTCTTCCTCTGG
PLLP-R (ms)	AGAGAACCGTGGCAGCGACAAA
MPC1-F (ms)	CTCCAGAGATTATCAGTGGGCG
MPC1-R (ms)	GAGCTACTTCGTTTGTTACATGGC
UBE2Q2-F (ms)	CAGGAAGACTCAAAGGCAAGACC
UBE2Q2-R (ms)	CCTGCCTTGTAGCTCTGTGATC
TBC1D2B-F (ms)	TGGAACTCTCGGCTCTACGAAG
TBC1D2B-R (ms)	TTCAACCTGGCAGAGCTTGGCT
FAM207A-F (ms)	TAGTGCGTTGGTGCAGAGGCTG
FAM207A-R (ms)	TGTAACCATCGCTCACGGCGAA
CHKA-F (ms)	TTGGCGATGAGCCTCGGAAAGT
CHKA-R (ms)	GTGACCTCTCTGCAAGAATGGC

TXNIP-F (ms)	GTTGCGTAGACTACTGGGTGAAG
TXNIP-R (ms)	CTCCTTTTTGGCAGACACTGGTG
FGFR1OP2-F (ms)	TCTCGGCACATCCTTGAAGCAC
FGFR1OP2-R (ms)	TGCTGCCATCTCGGTGATTTGG
KCTD5-F (ms)	TGCCAGTGAAGCATGTGTACCG
KCTD5-R (ms)	GTAAGAGGAGCCAATGCTGACC
IPMK-F (ms)	CTTCACTCTGACAGCTACGAGAC
IPMK-R (ms)	GAATACTGGCGGCAATCGCATC
STXBP5-F (ms)	GCTGGTCATTCAGTTGGTGTGG
STXBP5-R (ms)	CTGGAGGTAGTCAACCATTGCG
MAN1A2-F (ms)	CGGTGGGTTTTCTGGTGTCAAG
MAN1A2-R (ms)	GGTAGAAGGTCATCGCCAGAGA
DIAPH3-F (ms)	TCAGCATCTCCTGCTCATTCGC
DIAPH3-R (ms)	GTGAAGTCAGGGTCCGTTCCAT
SH3GL1-F (ms)	AGTCAGAGGGTCTGTTGGGAGA
SH3GL1--R (ms)	CGATGTCCAGTGAGTCCTTCAC
CAB39L-F (ms)	CAGCAAGCCAGAGAACCTGAAAC
CAB39L-R (ms)	CTCCACGATAGGCTGCGTTTTG
CENPO-F (ms)	AAGATAGCCGCAGCACACCTAC
CENPO-R (ms)	CGTGAGGACATCGCAGAAGTCA
LEMD2-F (ms)	CTCTTCGAGGTTTAAGGCTGCG
LEMD2--R (ms)	CCACTTTGTCCACTGTAGTCGC
HAT1-F(ms)	GATGGAGCTACGCTCTTTGCGA
HAT1-R(ms)	GCCCTGACCTTGAAATGGAGTC
C1orf210-F(ms)	TCCTCTCCATTCTCATCGCGCT
C1orf210-R(ms)	AGGCTGGATGTAATTGTCCTCGA
msZeb1-F	ATGCTCTGAACGCGCAGC
msZeb1-R	AATCGGCGATCTTTGAGAGCT
msCDH1-F	CCATCTCAAGCTCGCGGATA
msCDH1-R	TCCAACGTGGTCACCTGGT
msCDH2-F	GCCATTGATGCGGATGATC
msCDH2-R	CCTGTACCGCAGCATTCCAT
msVimentin-F	TCCAAGCCTGACCTCACTGC
msVimentin-R	TTCATACTGCTGGCGCACAT
msRab22a-F	CAGCATTGTCGTTGCCATCGCA
msRab22a-R	CGCTGGTCTCTACAAAGATGGC
hsRab22a-F	GCACCAATGTACTATCGAGGGTC
hsRab22a-R	CATGCTGTCGAAGCTCTTTCACC

Supplementary Table 2: Complete antibody information

Gene	Vendor	Catalog No.	WB	ChIP	PLA
ZEB1	Santa Cruz	H-102, H-102X	1:500	5μg	1:200
ZEB1	Cell Signaling	3396			1:200
GFP	Santa Cruz	SC-9996	1:1000		
normal sheep IgG	Sigma	I8265		5μg	
normal mouse IgG	Santa Cruz	SC-2025			
normal rabbit IgG	Santa Cruz	SC-2027			
E-Cadherin	B&D	160182			
Flag	Sigma	F1804	1:1000		
MTA1	Cell Signaling	5647	1:1000		1:500
MTA2	Santa Cruz	Sc-9447	1:1000		1:200
MTA3	Santa Cruz	81325	1:1000		1:200
HDAC1	Cell Signaling	5356	1:1000		1:500
HDAC2	Cell Signaling	5113	1:1000		1:50
CHD3	Cell Signaling	4241	1:1000		1:500
CHD4	Abcam	ab72418	1:1000	5μg	1:200
Rab22	Santa Cruz	SC-390726	1:1000		
TBC1D2b	Santa Cruz	SC-398906	1:1000		
β-actin	Sigma	A1978	1:10000		

Supplementary Table 3: ChIP primer sequences

Gene	ChIP Primer Sequence
miR-200c,141- F	AGGGCTCACCAGGAAGTGT
miR-200c,142- R	AGATCCCTGGCTCCCATC
TBC1D2-F(hs) #1	GAGACTGCGGAGGGACGAG
TBC1D2-R(hs) #1	CCCAGGTGTCTCCCTTTGGG
TBC1D2-F(hs) #2	GGCAGCTTCCCAAAGGGAGA
TBC1D2-R(hs) #2	CGCCGTAACCTGGGTTTGC
TBC1D2-F(hs) #3	GAGACTGCGGAGGGACGAG
TBC1D2-R(hs) #3	CCCAGGTGTCTCCCTTTGGG
KCNK1-F(hs) #1	ACCTGCTGGTTCCCGTAACA
KCNK1-R(hs) #1	GTGAGGCCAAGAGAGGTGCT
KCNK1-F(hs) #2	TCCTGGTGCTGGGCTACTTG
KCNK1-R(hs) #2	GCACTCGTGCTCCTCCAAGA
KCNK1-F(hs) #3	GAGCACGAGTGCCTGTCTGA
KCNK1-R(hs) #3	GTTGCTGAGCACCGACACG
EPS8L2-F(hs) #1	GACGGAGGCTCCCAAGAAGG
EPS8L2-R(hs) #1	ACTCACGGCAGCACATGGA
EPS8L2-F(hs) #2	TAAGGGAAGTGACTCTGCCC
EPS8L2-R(hs) #2	AGGCCTCGAGCTCTTCCTT
EPS8L2-F(hs) #3	GGGGCTGCCACAAAGAAAA
EPS8L2-R(hs) #3	ACATACCTGCCCCAGGTGA
SEMA3F-pr ChIP- hs F	GGCGTATGGATGTGTGGATGA
SEMA3F-pr ChIP- hs R	TATGAGAGCACCCACCCAGAAC
SEMA3F-neg ChIP- hs F	CCCTACAGTTCCAGCAGCCC
SEMA3F-neg ChIP- hs R	CCACCAACCCAGACCCTGAT
DNM1-F(hs) ChIP	AGACCCAACCCATTGACAAA
DNM1-R(hs) ChIP	GGCATCATGGGTGTCGTAGT
PTPN6-F(hs) ChIP	TCCATTTACCTCCGCTGAAC
PTPN6-R(hs) ChIP	GATTCTCACCCTTTGCTTGC
TXNIP-F(hs) ChIP	CAGCCCCAAACCTGAAAGTA
TXNIP-R(hs) ChIP	AGAGCCTGTCGTTATTCCTG
PTPN6-F(hs) ChIP #2	GCTTGGGGTATGAAGGTTTG
PTPN6-R(hs) ChIP #2	CAGCGGAGGTAAATGGAAAA
TBC1D2b-F(hs) ChIP #1	GGGTCAGTTGCCTTCGTG
TBC1D2b-R(hs) ChIP #1	GAAATAGACCATTGCTTCATCC
TBC1D2b-F(hs) ChIP #2	GCCCTGGTAGCTGAAGCA
TBC1D2b-R(hs) ChIP #2	CGCCGCTGCTACCTTTACT
