# Supplementary material for: ZEB1/NuRD complex suppresses TBC1D2b to stimulate E-cadherin internalization and promote metastasis in lung cancer
Source: Nat Commun. 2019 Nov 12;10:5125. doi: 10.1038/s41467-019-12832-z (PMC6851102; doi:10.1038/s41467-019-12832-z)
Supplement: Supplementary file 3 — Description of Additional Supplementary Files [file 41467_2019_12832_MOESM3_ESM.docx]

**Description of Supplementary Files**

**File Name:** Supplementary Data 1

**Description:** High confidence ZEB1 interactome.

**File Name:** Supplementary Data 2

**Description:** RSA values and rank scores for the Epigenome shRNA dropout screen.

**File Name:** Supplementary Data 3

**Description:** ZEB1/CHD4 binding sites by ChIP-seq from ENCODE.
