# Supplementary material for: ZEB1/NuRD complex suppresses TBC1D2b to stimulate E-cadherin internalization and promote metastasis in lung cancer
Source: Nat Commun. 2019 Nov 12;10:5125. doi: 10.1038/s41467-019-12832-z (PMC6851102; doi:10.1038/s41467-019-12832-z)
Supplement: Supplementary file 7 — Reporting Summary [file 41467_2019_12832_MOESM7_ESM.pdf]

nature research

Corresponding author(s): *Double-blind peer review submissions: write DBPR and your manuscript number here instead of author names.*

Last updated by author(s): Sep 10, 2019

Reporting Summary

Nature Research wishes to improve the reproducibility of the work that we publish. This form provides structure for consistency and transparency in reporting. For further information on Nature Research policies, see Authors & Referees and the Editorial Policy Checklist .

Statistics

For all statistical analyses, confirm that the following items are present in the figure legend, table legend, main text, or Methods section.

n/a	Confirmed	
☐	☒	The exact sample size (*n*) for each experimental group/condition, given as a discrete number and unit of measurement
☐	☒	A statement on whether measurements were taken from distinct samples or whether the same sample was measured repeatedly
☐	☒	The statistical test(s) used AND whether they are one- or two-sided<br>*Only common tests should be described solely by name; describe more complex techniques in the Methods section.*
☒	☐	A description of all covariates tested
☒	☐	A description of any assumptions or corrections, such as tests of normality and adjustment for multiple comparisons
☐	☒	A full description of the statistical parameters including central tendency (e.g. means) or other basic estimates (e.g. regression coefficient) AND variation (e.g. standard deviation) or associated estimates of uncertainty (e.g. confidence intervals)
☐	☒	For null hypothesis testing, the test statistic (e.g. *F*, *t*, *r*) with confidence intervals, effect sizes, degrees of freedom and *P* value noted<br>*Give P values as exact values whenever suitable.*
☒	☐	For Bayesian analysis, information on the choice of priors and Markov chain Monte Carlo settings
☒	☐	For hierarchical and complex designs, identification of the appropriate level for tests and full reporting of outcomes
☒	☐	Estimates of effect sizes (e.g. Cohen's *d*, Pearson's *r*), indicating how they were calculated

Our web collection on statistics for biologists contains articles on many of the points above.

Software and code

Policy information about availability of computer code

Data collection	No software was used in the present study.
Data analysis	No software was used in the present study.

For manuscripts utilizing custom algorithms or software that are central to the research but not yet described in published literature, software must be made available to editors/reviewers. We strongly encourage code deposition in a community repository (e.g. GitHub). See the Nature Research guidelines for submitting code & software for further information.

Data

Policy information about availability of data

All manuscripts must include a data availability statement. This statement should provide the following information, where applicable:

- Accession codes, unique identifiers, or web links for publicly available datasets
- A list of figures that have associated raw data
- A description of any restrictions on data availability

The ChIP-seq data referenced during the study are available in a public repository from the ENCODE website. The source data underlying Supplementary Tables 1, 2, and 3 are provided as a Source Data file. All the other data supporting the findings of this study are available within the article and its supplementary information files and from the corresponding author upon reasonable request. A reporting summary for this article is available as a Supplementary Information file.

Field-specific reporting

Please select the one below that is the best fit for your research. If you are not sure, read the appropriate sections before making your selection.

Life sciences study design

All studies must disclose on these points even when the disclosure is negative.

Sample size	The smallest number of mice possible we reused to carry out the experiments. Because of the potential variability of the phenotypes in vivo, it is difficult to provide exact numbers. But we always assume that a few mice will die of natural causes (premature illness or infection). Based on our long-term experience with mouse models of human lung cancer, we estimated:<br>Overexpression study- We injected 6 wildtype mice per group (control and TBC1D2b).<br>Knockdown study- We selected the shRNA with the most efficient knockdown and injected 6 wildtype mice per group (control and TBC1D2b).
Data exclusions	Data was excluded from the analysis depending on whether the mice died of natural causes or if implantation of tumor failed.
Replication	Six mice were injected per experimental group to confirm reproducibility of experimental findings.
Randomization	Mice were distributed randomly in all data collection and analysis.
Blinding	Blinding was not relevant to this publication.

Reporting for specific materials, systems and methods

We require information from authors about some types of materials, experimental systems and methods used in many studies. Here, indicate whether each material, system or method listed is relevant to your study. If you are not sure if a list item applies to your research, read the appropriate section before selecting a response.

Materials & experimental systems

n/a	Involved in the study	
☐	☒	Antibodies
☐	☒	Eukaryotic cell lines
☒	☐	Palaeontology
☐	☒	Animals and other organisms
☒	☐	Human research participants
☒	☐	Clinical data

Methods

n/a	Involved in the study	
☒	☐	ChIP-seq
☒	☐	Flow cytometry
☒	☐	MRI-based neuroimaging

Antibodies

Antibodies used	ZEB1-Santa Cruz H102/H102X<br>ZEB1 Cell Signaling 3396<br>GFP Santa Cruz SC-9996<br>normal mouse IgG Santa Cruz SC-2025<br>normal rabbit IgG Santa Cruz SC-2027<br>E-Cadherin B&D 160182<br>Flag Sigma F1804<br>MTA1 Cell Signaling 5647<br>MTA2 Santa Cruz Sc-9447<br>MTA3 Santa Cruz 81325<br>HDAC1 Cell Signaling 5356<br>HDAC2 Cell Signaling 5113<br>CHD3 Cell Signaling 4241<br>CHD4 Abcam ab72418<br>GFP Santa Cruz SC-9996<br>Rab22 Santa Cruz SC-390726<br>TBC1D2b Santa Cruz SC-398906<br>B-actin Sigma A1978
Validation	Validation of most antibodies was performed by overexpression or knockdown of the gene of interest. Mass spectrometry was performed to confirm specificity of ZEB1 antibody (data not shown). Additionally, immunofluorescence was performed to confirm antibody specificity and optimize dilution prior to performance of PLA assay.

Eukaryotic cell lines

Policy information about cell lines

Cell line source(s)	Human lung cancer cell lines H157, H1299 and H358 were obtained from the National Cancer Institute (NCI-H series) or the Hamon Center for Therapeutic Oncology Research, University of Texas Southwestern Medical Center (HCC series). Cell lines from the KP mice were derived and maintained as previously described. HEK/293 Flp-In T-Rex were provided by the Raught laboratory (University of Toronto).
Authentication	All murine NSCLC cell lines were authenticated as previously described in Gibbons et al. Human lines were authenticated prior to obtaining from NCI and HCC series.
Mycoplasma contamination	All cell lines were tested for mycoplasma every three months. Additional mycoplasma detection was performed upon generation of new cell lines or prior to in vivo implantation.
Commonly misidentified lines (See ICLAC register)	None of the lines utilized in this study are listed under the ICLAC register of commonly misidentified lines.

Animals and other organisms

Policy information about studies involving animals; ARRIVE guidelines recommended for reporting animal research

Laboratory animals	Three month old SV129 mice were utilized for in vivo experiments.
Wild animals	This study did not involve wild animals.
Field-collected samples	This study did not involve field-collection samples.
Ethics oversight	All animal experiments were reviewed and approved by the Institutional Animal Care and Use Committee at The University of Texas M.D. Anderson Cancer Center.

Note that full information on the approval of the study protocol must also be provided in the manuscript.
